# Proteomic analysis reveals the molecular mechanism of Astragaloside in the treatment of non-small cell lung cancer by inducing apoptosis

**DOI:** 10.1186/s12906-023-04305-0

**Published:** 2023-12-15

**Authors:** Jiaqi Liu, Yan Sun, Wenjing Chen, Lingling Deng, Mengmeng Chen, Jingcheng Dong

**Affiliations:** 1 Department of Integrative Medicine, Huashan Hospital, Fudan University, 12 Middle Urumqi Road, Shanghai, 200040 China; 2 Institutes of Integrative Medicine, Fudan University, Shanghai, China

**Keywords:** Astragaloside III (AS III), Non-small cell Lung cancer, Proteomics, Apoptosis, MAPKs pathway

## Abstract

**Background:**

Astragaloside III (AS III), a saponin-like metabolite derived from the traditional Chinese medicine *Astragali Radix*, has been shown to be effective in the treatment of cancer and heart failure, and a variety of digestive disorders. However, its molecular mechanism in the treatment of non-small cell lung cancer (NSCLC) is unknown.

**Methods:**

Human lung cancer A549 cells and NCI-H460 cells and a normal human lung epithelial cell BEAS-2B were treated with different concentrations of AS III. CCK-8 and EdU staining were used to determine the anti-proliferative effects of AS III in vitro. Quantitative proteomic analysis was performed on A549 cells treated with the indicated concentrations of AS III, and the expression levels of apoptosis-related proteins were examined by Western blotting.

**Results:**

AS III treatment significantly inhibited proliferation and increased apoptosis in A549 and H460 cells and modulated functional signaling pathways associated with apoptosis and metabolism. At the molecular level, AS III promoted a reduction in the expression of ANXA1 (*p* < 0.01), with increased levels of cleaved Caspase 3 and PARP 1. In addition, AS III treatment significantly decreased the LC3-I/LC3-II ratio. The results of experiment in vitro showed that AS III promoted NSCLC apoptosis by down-regulating the phosphorylation levels of P38, JNK, and AKT (*p* < 0.01), inhibiting the expression of Bcl-2 (*p* < 0.01), and up-regulating the expression of Bax (*p* < 0.01).

**Conclusion:**

These findings provide a mechanism whereby AS III treatment induces apoptosis in NSCLC cells, which may be achieved in part via modulation of the P38, ERK and mTOR signaling pathways.

**Supplementary Information:**

The online version contains supplementary material available at 10.1186/s12906-023-04305-0.

## Introduction

Lung cancer has the highest worldwide morbidity and mortality rates of any malignant neoplasm [1, 2]. Based on data from the World Health Organization, 2.1 million cases are newly diagnosed each year [3, 4]. Lung cancer accounts for 18.4% of all cancer deaths [5], and non-small cell lung cancer (NSCLC) accounts for 85% of lung cancers, with a 5-year survival of only 15% [6]. NSCLC can be further divided into three subtypes according to histological features, with lung adenocarcinoma being the most common subtype [7, 8]. Thus, NSCLC has a poor prognosis and a serious impact on human health and socioeconomic development [9].

The dried root of the perennial legume *Astragalus membranaceus* (Fisch.) Bge. var. *mongholicus* (Bge.) Hsiao or *Astragalus membranaceus* (Fisch.) Bge. is known in traditional Chinese medicine as *Astragali Radix*, or “Huang Qi” in Chinese, which has therapeutic efficacy for colorectal cancer, diabetes, nephritis and other diseases [10–12]. Modern pharmacological studies have confirmed that Astragalus saponins can protect against cerebral ischemia-reperfusion injury by activating Nrf2 and inhibiting NLRP3 inflammasome-mediated febrile effects [13]. In an in vivo mouse model, Astragalus saponin inhibited GRP and modulated calpain expression in human colon cancer, resulting in significant inhibition of tumor growth [14]. In addition, Astragalus extracts obtained by the water extraction-ethanol supernatant method exhibited dose-dependent anti-proliferative activity towards MCF-7, SK-BR-3 and other breast cancer cells, and the potential mechanism of action involved the induction of apoptosis through modulation of the PI3K/AKT/mTOR signaling pathway [15]. In a natural aging model, aqueous extract of Astragalus also significantly prolonged the lifespan of *Drosophila melanogaster* through an antioxidant mechanism [16]. Many additional studies provide evidence supporting the inhibitory effect of Astragalus saponin on the proliferation and metastasis of cancer cell lines, including hepatocellular carcinoma, colorectal carcinoma, and gastric adenocarcinoma cells [17–19].

ANXA1 is an endogenous immunomodulatory protein that plays a role in tumor development, including cell proliferation, apoptosis, metastasis, and invasion [20]. ANXA1 has recently been recognized as a valuable diagnostic or prognostic biomarker, and is often differentially expressed in tumor and normal tissue samples. Studies have shown that ANXA1 is highly expressed in lung adenocarcinoma [21], hepatocellular carcinoma [22], and pancreatic cancer [23], and is associated with poor prognosis [21, 22] and low metastatic survival [22, 24]. However, ANXA1 is highly expressed in nasopharyngeal carcinoma [25] and prostate cancer [26].

Astragaloside III (AS III) is a saponin-like metabolite of the Astrogalus species *Astragali Radix* (Supplementary Fig. 1). Recently, *Astragali Radix* saponin-like extracts have been shown to have therapeutic effects in treating NSCLC [27, 28]. In our study, the proliferative viability of human lung cancer A549 cells and NCI-H460 cells was evaluated in vitro using CCK-8 assay and EdU. And we obtained the differential proteins after AS III treatment using proteomics, and used flow cytometry to study the apoptosis of A549 cells and NCI-H460 cells. The core target proteins and potential pro-apoptotic molecular mechanisms of AS III were predicted. Our findings provide a theoretical basis for the application of *Astragali Radix* saponin-like extracts in clinical practice.

## Materials and methods

### Cell culture and treatment

The human lung cancer epithelial cell line A549, cell line NCI-H460 and normal human lung epithelial cell line BEAS-2B were purchased from the Cell Bank of the Shanghai Institutes for Biological Sciences, Chinese Academy of Sciences, at the Institute of Integrative Medicine, Huashan Hospital, Fudan University. A549 and H460 cells were cultured in RPMI-1640 (Kaiji, Nanjing, China), and BEAS-2B cells were cultured in DMEM (Gibco, California, USA), both media were supplemented with 10% fetal bovine serum (Gibco, California, USA). AS III with > 98% purity (Puyi, Nanjing, China) was dissolved in DMSO (Merck Sigma, St. Louis Missouri, USA) to obtain a stock solution, which was stored at -80 °C. As a positive control drug, DDP (Merck Sigma, St. Louis Missouri, USA) was dissolved in sterile saline and stored at 4 °C.

### CCK-8 assays

A549, H460 and BEAS-2B cells cells in optimal growth conditions were prepared as a cell solution and inoculated in 96-well cell culture plates (100 µL per well). The cells were fully adhered after 24 h of incubation. The medium was aspirated and replaced with gradient concentrations (0, 25, 50, 100, 150, 200, 250, 300, 350, 400 µmol/L) of AS III-containing medium (six replicates each). After 24 h, 10 µL of CCK-8 color development solution (Beyotime, Shanghai, China) was added per well, and after 1–3 h, the OD value at 450 mm was measured.

### The 5-ethynyl-2′-deoxyuridine (EdU) incorporation assay

A549 and H460 cells were inoculated into 6-well plates with 6 × 10^5^ cells/well and incubated at 37 °C, 5% CO_2_. Cells were cultured with EdU for 2 h according to the EdU Cell Proliferation Kit (C0085S, Beyotime, Shanghai, China), then fixed with 4% paraformaldehyde for 30 min, and the nuclei were stained with Hoechst 33,342 (5 µg/mL, Beyotime, Shanghai, China), and images were captured under a fluorescence microscope (Olympus, Tokyo, Japan).

### Apoptosis assay

A549 and H460 cells in optimal growth conditions were prepared into cell solutions and inoculated in 6-well cell culture plates. The cells were fully attached after 24 h of incubation. After treating the cells with different concentrations of AS III for 24 h, A549 and H460 cells were harvested with PBS. Samples were analyzed by flow cytometer (FACSCalibur, California, USA) after incubation with PI and Annexin V-Alexa Fluor (Thermo Fisher Scientific, USA) for 20 min at room temperature.

### Protein preparation

The cells were washed three times with pre-cooled PBS and centrifuged at 6000 g for 6 min and then 6000 g for 4 min. The supernatant was removed, and the cells were lysed by resuspension in RIPA containing protease inhibitors (1:100 dilution) and phosphatase inhibitors (1:200 dilution), with 2 to 3 rounds of sonication using an ultrasonic disruptor. The extract was centrifuged at 13,000 rpm for 10 min at 4 °C in a frozen low-speed centrifuge, and the supernatant was collected. The protein concentration was determined using a BCA kit (Thermo Fisher Scientific, USA).

### Liquid chromatography and tandem mass spectrometry (LC-MS/ MS) analysis

All analyses were performed using a Q-Exactive mass spectrometer (Thermo Fisher Scientific, USA) equipped with a Nanospray Flex source (Thermo Fisher Scientific, USA). The samples were loaded and separated on a C18 column (15 cm × 75 μm) with an EASY-nLCTM 1200 system (Thermo Fisher Scientific, USA). The flow rate was 300nL/min, and the total run was 75 min (0 ~ 40 min, 2–28% B; 40 ~ 50 min, 28–42% B; 50 ~ 55 min, 42–90% B; 55 ~ 75 min, 90% B; mobile phase A = 0.1% FA in water and B = 0.1% FA in ACN).

Full MS scans were acquired in the range of 350–1500 m/z with a resolution of 60,000. The AGC target value was set at 3e6. The ten most intense MS peaks were fragmented with higher-energy collisional dissociation with a normalized collision energy of 32. MS/MS spectra were obtained at a resolution of 45,000 with an AGC target value of 2e5 and a max injection time of 80ms. The Q-E dynamic exclusion was set for 30 s and run under the positive mode.

### Bioinformatics analysis

For screening, foldchange ≥ 2 and ≤ 1/2 were set as the threshold, and the differentially expressed proteins (DEPs) that passed the threshold were functionally annotated and enriched.

### Western blot

Proteins (8 µL per well) were separated by electrophoresis using 12% SDS-PAGE and then transferred to PVDF membranes. The membranes were blocked with 5% skim milk for 2 h at room temperature. After 3 washes in TBST for 5 min each, the membranes were placed in incubation bags with primary antibody overnight at 4 °C. The primary antibodies were as follows: Anti-α-Tubulin (ab184970, Abcam, USA), anti-Bcl-2(ab182858, Abcam, USA), anti-Bax(ab32503, Abcam, USA), anti-LC3 (14600-1-AP, Proteintech Genomics, USA), anti-P62 (18420-1-AP, Proteintech Genomics, USA), anti-Beclin-1(11306-1-AP, Proteintech Genomics, USA), anti-ERK1/2 (ab17942, Abcam, USA), anti-P-ERK1/2 (ab201015, Abcam, USA), anti-Akt (ab38449, Abcam, USA), anti-P-Akt (ab38449, Abcam, USA), anti-ANXA1 (21990-1-AP, Proteintech Genomics, USA), anti-Caspase-3 (19677-1-AP, Proteintech Genomics, USA), anti-cleaved Caspase-3 (25128-1-AP, Proteintech Genomics, USA), anti-PARP1 (13371-1-AP, Proteintech Genomics, USA), anti-cleaved PARP1(ab4830, Abcam, USA), anti-mTOR (66888-1-lg, Proteintech Genomics, USA), and anti-P-mTOR (67778-1-lg, Proteintech Genomics, USA). After 3 washes with TBST for 10 min each, the secondary antibody (SA00001-2, Proteintech Genomics, USA) was applied, and the samples were incubated for 2 h at room temperature. ECL chemiluminescence reagent (Tanon Scientific, China) was applied, and the grayscale values of the proteins were analyzed using ImageJ software.

### Statistical analysis

The results of this study were expressed as mean ± SD (standard deviation). GraphPad Prism 9 (GraphPad Software Inc., USA) software was used for statistical analysis. Differences with *P*-value < 0.05 were considered statistically significant. * indicates *P*-value < 0.05, ** indicates *P*-value < 0.01, and *** indicates *P*-value < 0.001.

### Data Availability

Data that support the findings of this study are available from the corresponding author on request.

## Results

### Cell viability of A549, H460, and BEAS-2B cells treated with AS III

**Fig. 1:**
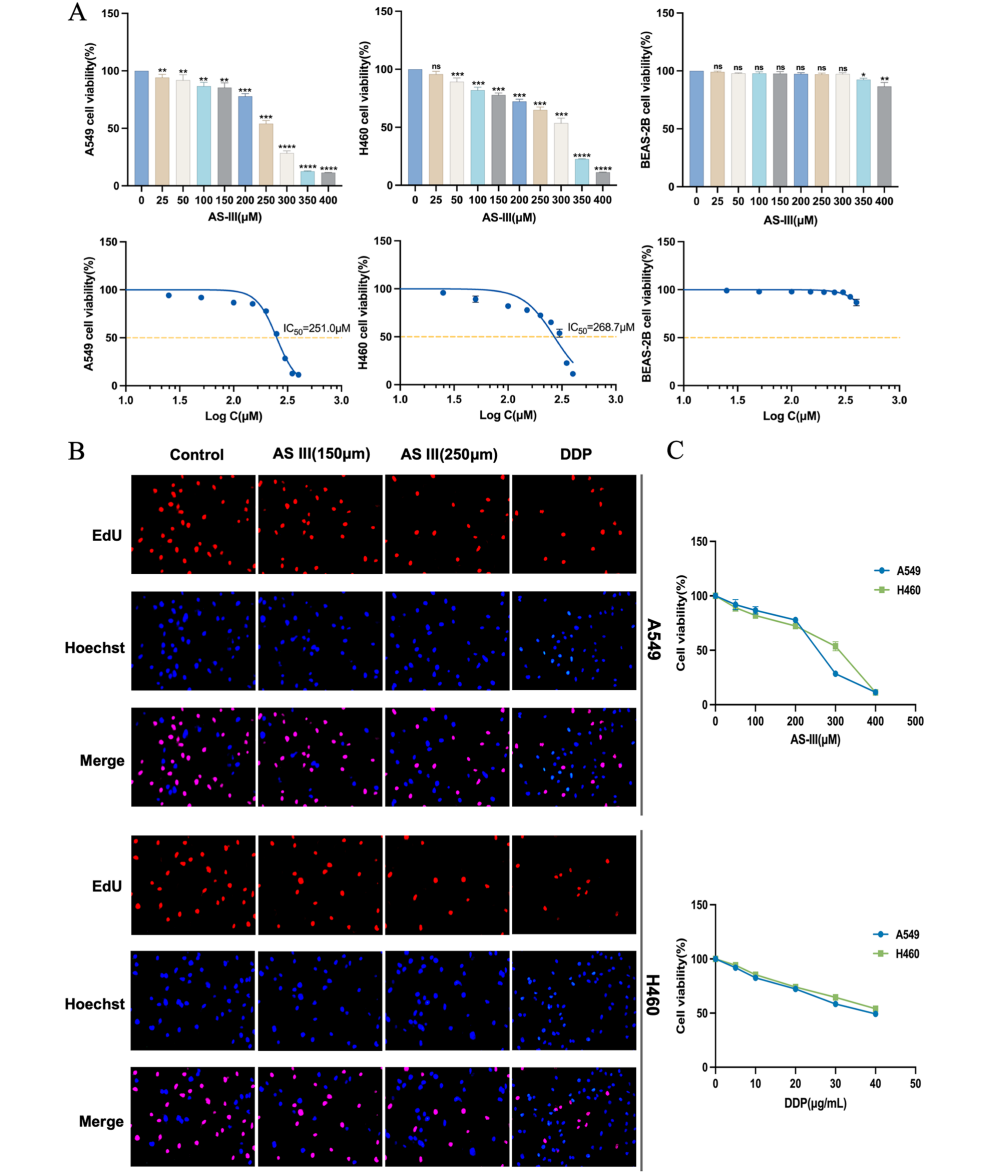
Effect of AS III on cell proliferation viability. (**A**) Cell viability and IC_50_ curves of A549, H460 and BEAS-2B cells treated with different concentrations of AS III for 24 h (values represent mean + SD of triplicates. *P < 0.05, **P < 0.01, *** *P* < 0.001). (**B**) Cells were treated with the indicated concentrations of AS III (150 and 250 µmol/L) and DDP (40 µg/mL) for 24 h, followed by EdU staining. (**C**) A549 and H460 cells were treated with different concentrations of AS III (0, 50, 100, 200, 300, and 400 µmol/L) and DDP (0, 5, 10, 20, 30, and 40 µg/mL) for 24 h, and cell viability was determined by CCK-8

To investigate the role of AS III in lung carcinogenesis and development, we seeded the cells in 96-well plates and treated them with the indicated concentrations(0, 25, 50, 100, 150, 200, 250, 300, 350, 400 µmol/L). The effect of AS III on the viability of A549, H460, and BEAS-2B cells in vitro was detected by CCK-8. The results showed that AS III significantly reduced the viability of A549 and H460 cells in a dose-dependent manner (*P* < 0.05), and there was no significant viability change in BEAS-2B at doses lower than 300 µmol/L(Fig. 1A). The IC_50_ values of A549 and H460 cells were 251.0 and 268.7 µmol/L. It is indicated that AS III could significantly reduce the viability of A549 and H460 cells under appropriate dosage conditions within 24 h, but had no significant effect on BEAS-2B cells. As a positive control drug, we examined the effects of DDP on the viability and proliferation of A549 and H460 cells using CCK-8. As shown(Fig. 1C), the semi-inhibitory concentrations of DDP treated A549 cells for 24 h were 39.45 µg/mL, and the semi-inhibitory rate was not reached by treating H460 cells for 24 h. Similar results were obtained in the EdU merging experiment(Fig. 1B).

Overall, the dose of 250 µmol/L significantly reduced the viability of NSCLC cells, especially on A549 cells, but it had no significant effect on BEAS-2B normal human lung epithelial cells. Therefore, A549 cells treated with a dose of 250 µmol/L were selected for proteomic analysis to continue the study of the potential mechanism of action of AS III in NSCLC treatment.

### AS III treatment alters protein expression in A549 cells

**Fig. 2:**
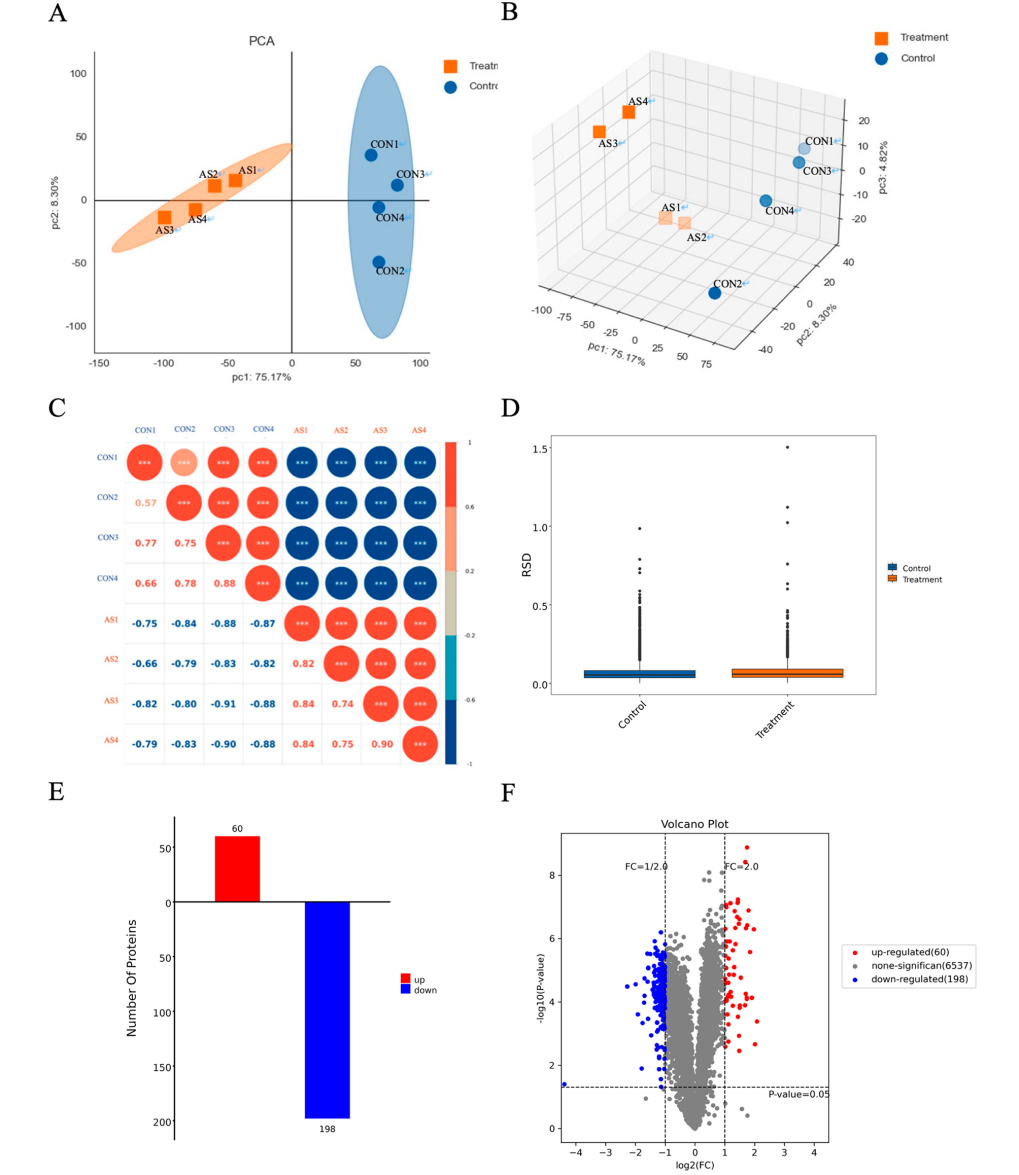
Mass spectrometry analysis of differentially expressed proteins in AS III-treated A549 cells. (**A-B**) Two-dimensional and three-dimensional maps of Credible Protein Principal component analysis (PCA). (**C**) Pearson correlation coefficient analysis. An absolute value close to 1 indicates a stronger linear correlation. (**D**) Relative standard deviation (RSD) distribution box line chart. (**E**) Distribution of differentially expressed proteins (DEPs). (**F**)Volcano plot of DEPs. Values represent mean + SD of duplicates

Based on the CCK-8 assay results, we performed quantitative proteomics of duplicate A549 cell samples using 250 µmol/L AS III, with principal component analysis, Pearson correlation coefficients and relative standard deviation to assess the degree of correlation between samples (Fig. 2A-D). According to a false discovery rate < 0.01, 65,027 peptides and 7068 quantifiable proteins were identified as potential DEPs. Using Foldchange ≥ 2 or ≤ 1/2, and *P*-value < 0.05 as cutoff criteria, 60 up-regulated and 198 down-regulated proteins were identified in AS III-treated A549 cells compared to untreated cells (Fig. 2E-F) (Supplementary Tables 1–2). Among these, 31 proteins were associated with apoptosis (Supplementary Table 3).

### Functional enrichment analysis suggests that AS III modulates the expression of apoptosis-associated proteins

**Fig. 3:**
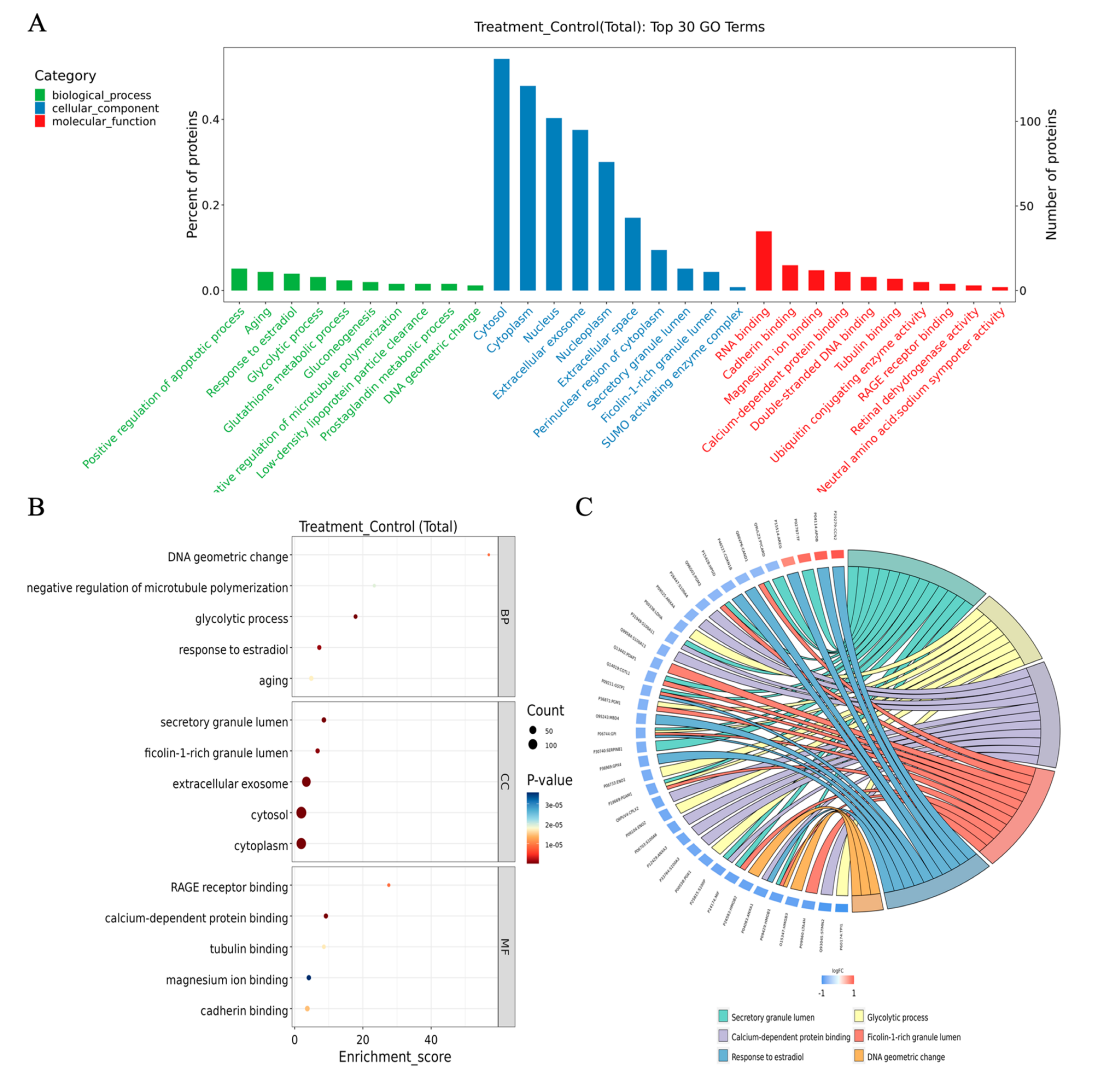
GO enrichment analysis of AS III-modulated proteins. (**A**) Gene Ontology (GO) enrichment plots of DEPs in Biological Process (BP), Cellular Component (CC) and Molecular Function (MF) categories. (**B**) GO annotations for 258 dysregulated proteins in BP (green), CC (blue) and MF (red) categories. Terms in the same category were sorted according to *P*-value. (**C**) GO enrichment chord diagram. Protein-gene names are shown on the left, and the selected GO term is shown on the below

To further identify the functions of the DEPs, we performed functional enrichment analysis. In GO analysis of Cellular Components, nearly half of the DEPs were located in the cell membrane and cytoplasm, and the rest were mainly located in the nucleus and extracellular bodies. For the Biological Processes, most of the identified proteins were closely associated with “apoptotic process” and “positive regulation of senescence” (Fig. 3A-C).

**Fig. 4:**
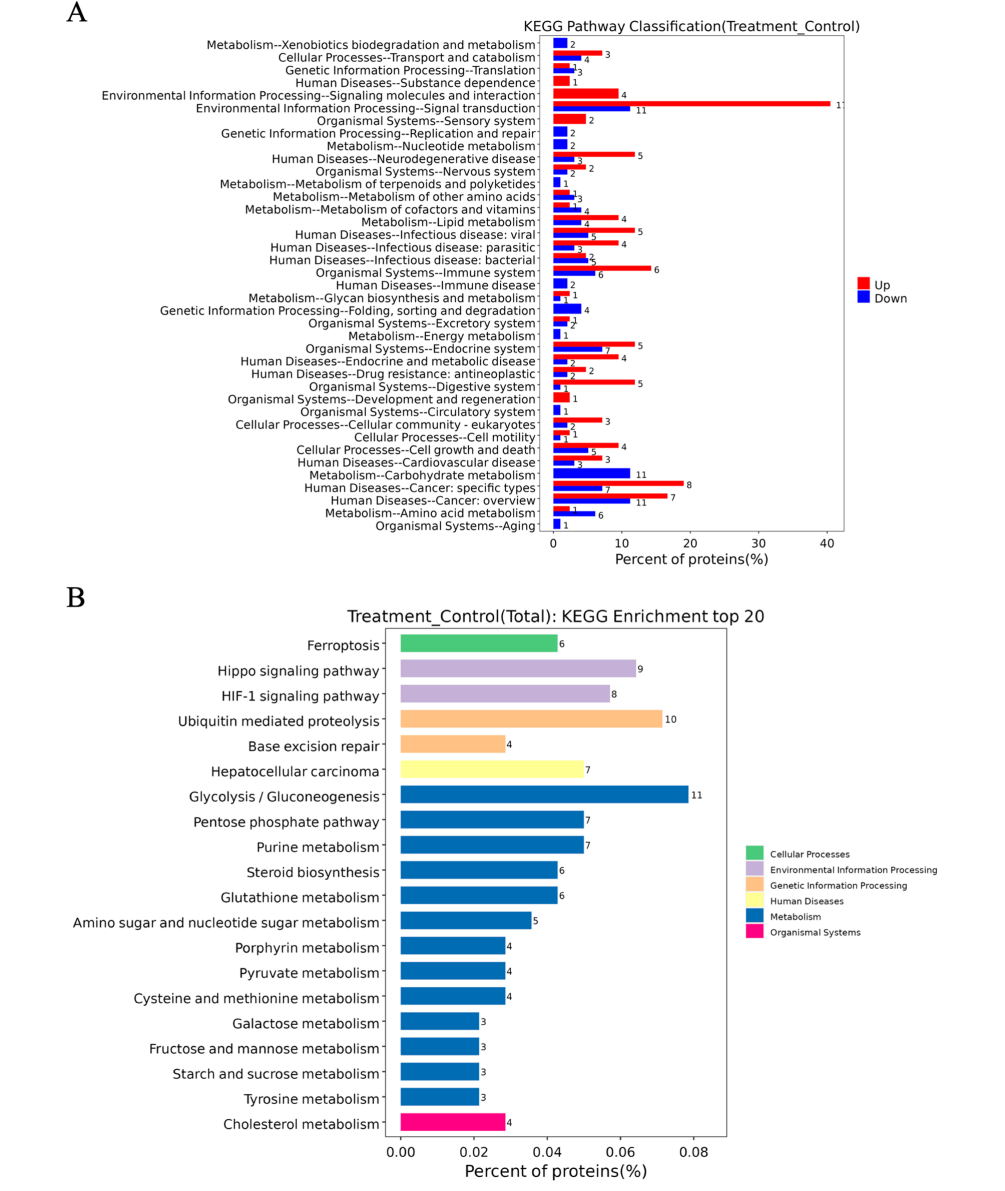
KEGG enrichment analysis of AS III-modulated proteins. The horizontal axis is the ratio of DEPs annotated for each metabolic pathway to the total number of all DEPs (%). (**A**) The distribution of up-regulated (down-regulated) DEPs at the KEGG Level2 level. (**B**) The distribution of DEPs at the KEGG Level3 level

Additionally, KEGG pathway analysis revealed that the DEPs were significantly enriched in the Steroid biosynthesis (hsa00100), Pentose phosphate pathway (hsa00030), and Glycolysis/Gluconeogenesis (hsa00010) (Fig. 4A). As seen from the distribution of DEPs at the KEGG Level3 (Fig. 4B), up-regulated and down-regulated pathways included Glycolysis/Gluconeogenesis (hsa00010), Ubiquitin mediated proteolysis (hsa04120), and Hippo signaling pathway (hsa04390). These findings further support a role for AS III in inducing apoptosis in NSCLC cells and indicate potential contributing mechanisms.

### Selection and validation of key target proteins associated with AS III-mediated apoptosis

**Fig. 5:**
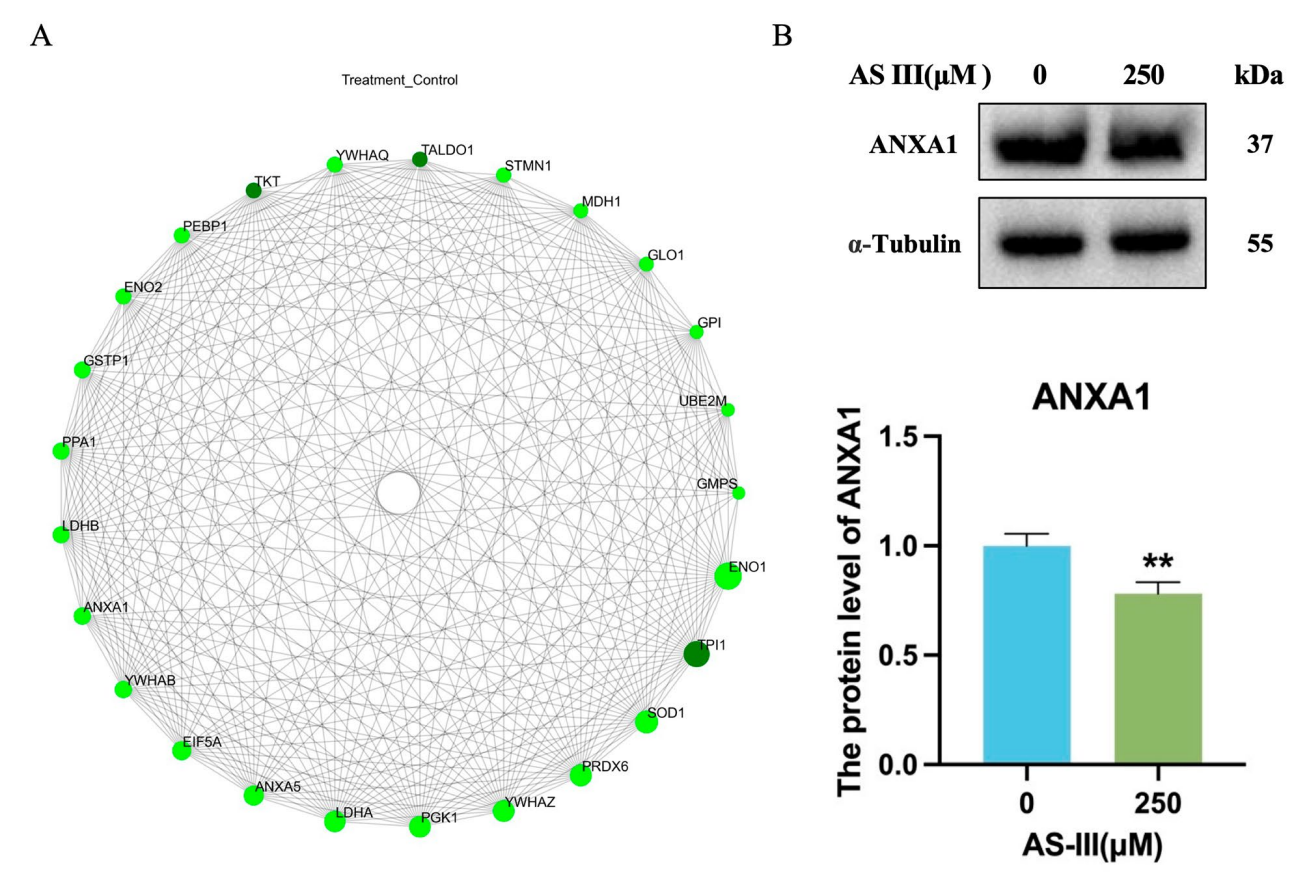
Selection and validation of ANXA1. (**A**) Protein-protein interaction (PPI) network. (**B**) Effect of AS III on the expression level of ANXA1 protein. Data are expressed as mean ± SD (n = 3). *P < 0.05 and **P < 0.01 compared to controls

To further investigate the functional relevance of these data, we performed protein network analysis and screened 25 proteins with the highest connectivity values (Fig. 5A). The results indicated that ENO1, TPI1 and ANXA1 were key proteins for maintaining homeostasis and stability that were downregulated by AS III treatment. ANXA1 has recently been shown to be involved in apoptosis induction in a variety of studies [29]. Therefore, we sought to validate the expression levels of ANXA1 in A549 cells by western blotting. The results verify that ANXA1 expression was reduced in the AS III-treated group compared with the control group (Fig. 5B) (*P* < 0.01).

### AS III induces cell apoptosis in A549, H460 cells

**Fig. 6:**
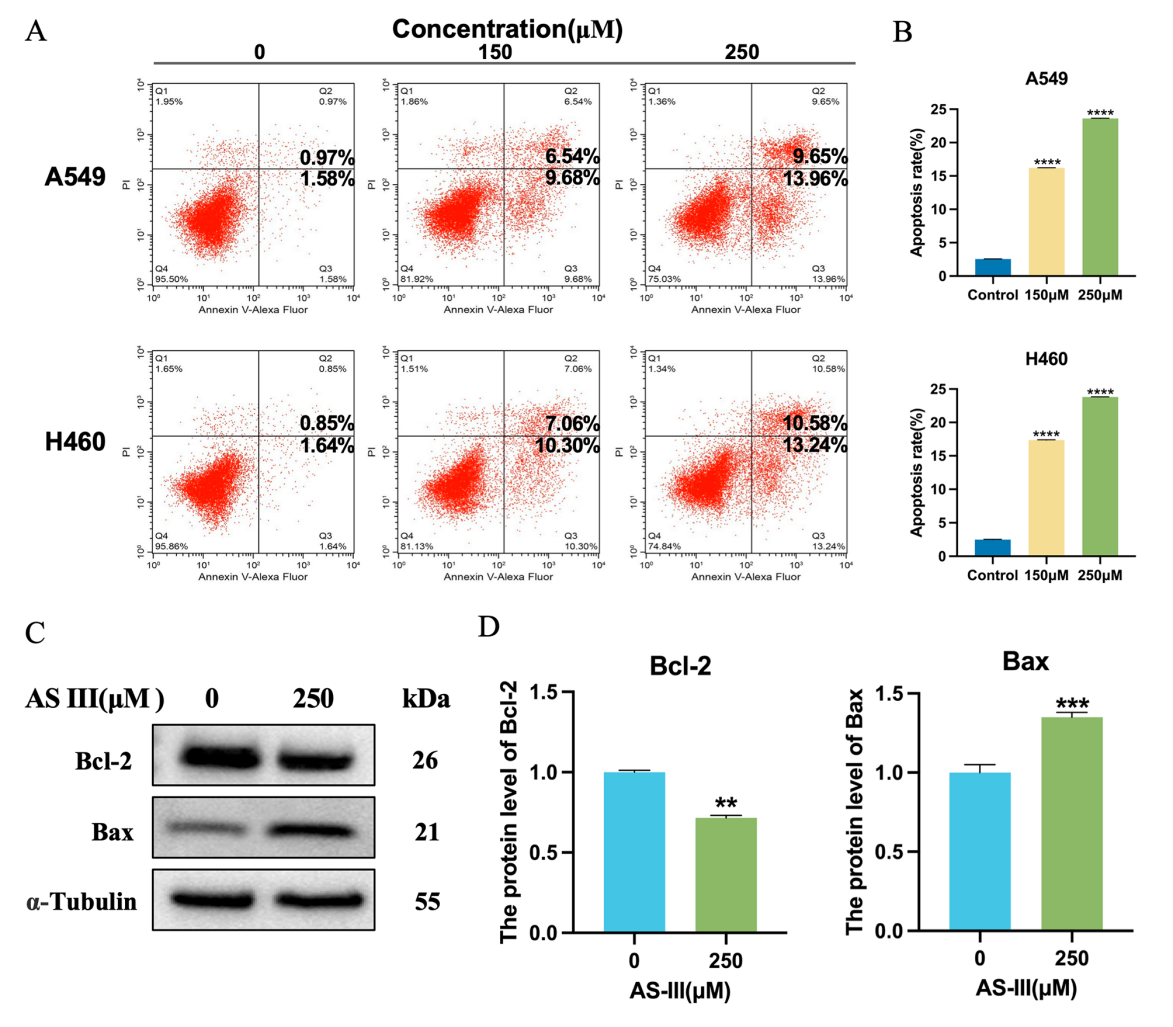
AS III induces apoptosis in lung cancer cells. (**A-B**) Annexin V-Alexa Fluor/PI staining assay for apoptosis detection using flow cytometry. A549 and H460 cells were treated with AS III for 24 h. (**C-D**) Effect of AS III (250µM) on Bcl-2 and Bax expression. Values are expressed as mean ± *S*.E.M. *P < 0.05, **P < 0.01, ***P < 0.001, compared to the AS III(0µM) group

To further confirm whether the reduction of cell viability in lung cancer cells after AS III treatment was associated with the promotion of apoptosis (Fig. 6A), flow cytometer was used to detect apoptosis. The results showed that the apoptosis rate of A549 and H460 cells treated with AS III for 24 h was increased (Fig. 6B) (*P* < 0.01). It was also observed that under the dosage within 250 µmol/L, not necrosis but apoptosis led to the significant reduction in cell viability of A549 and H460 cells. To further clarify the role of AS III in the apoptotic process, we also examined the expression levels of the apoptotic proteins Bcl-2 and Bax. Bcl-2 was significantly decreased (*P* < 0.01) and Bax was significantly increased (*P* < 0.01) after AS III intervention (Fig. 6C-D). The results indicated that AS III had a significant apoptosis-inducing effect.

**Fig. 7:**
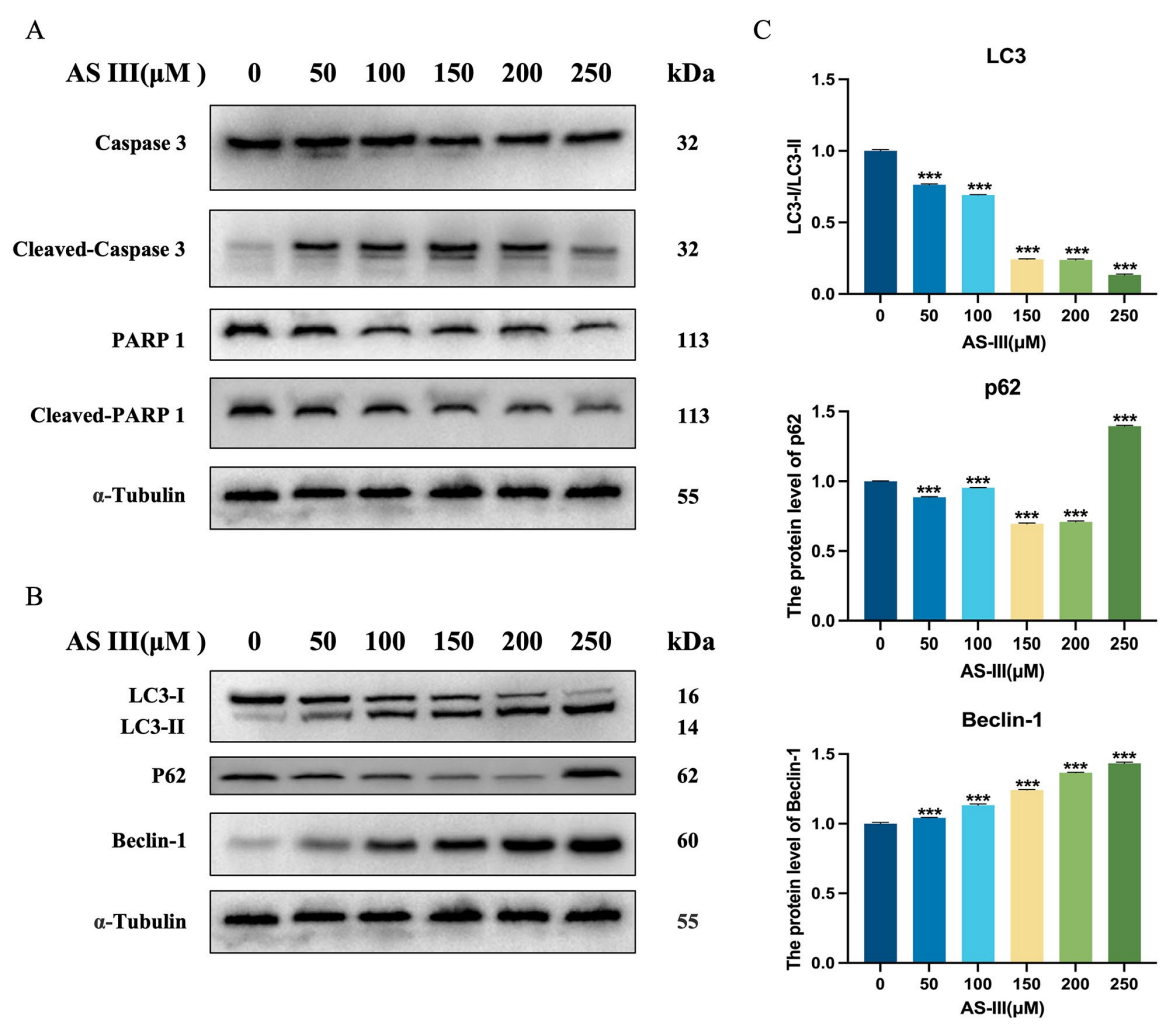
Effect of AS III on the expression of apoptosis and autophagy-associated proteins in A549 cells. (**A**) Effect of different concentrations of AS III on the expression of the intact and cleaved forms of Caspase 3 and PARP 1 (apoptotic markers). (**B-C**) Effect of different concentrations of AS III on the expression of LC3, Beclin-1 and P62 (autophagy markers). Values are expressed as mean ± *S*.E.M. *P < 0.05, **P < 0.01, ***P < 0.001, compared to the AS III(0µM) group

Given the well-established roles for Caspase 3 and its enzyme substrate poly ADP-ribose polymerase (PARP 1) in apoptosis [30], we further evaluated the protein expression levels of Caspase 3, PARP 1 and their cleaved (activated) forms. As shown in Fig. 7A, Caspase 3 and PARP 1 were activated after AS III treatment. We also evaluated the effects of AS III on the expression of autophagy marker proteins LC3, Beclin-1 and P62, each of which are recognized markers of autophagy [31, 32]. As shown in the figure (Fig. 7B), the expression of LC3-I decreased and LC3-II increased with increasing AS III concentration, AS III could down-regulate the ratio of LC3-I/LC3-II and activate Beclin-1.The expression of P62 also gradually decreased with increasing AS III concentration, though this trend was reversed at 250 µmol/L concentration (Fig. 7C). The occurrence of this phenomenon may be related to the blockage of autophagic flow and the stacking reaction of P62 as a selective autophagic substrate. Collectively, these results suggest that AS III modulates proteins associated with apoptosis and autophagy.

### AS III modulates the MAPK and AKT/mTOR signaling pathways

**Fig. 8:**
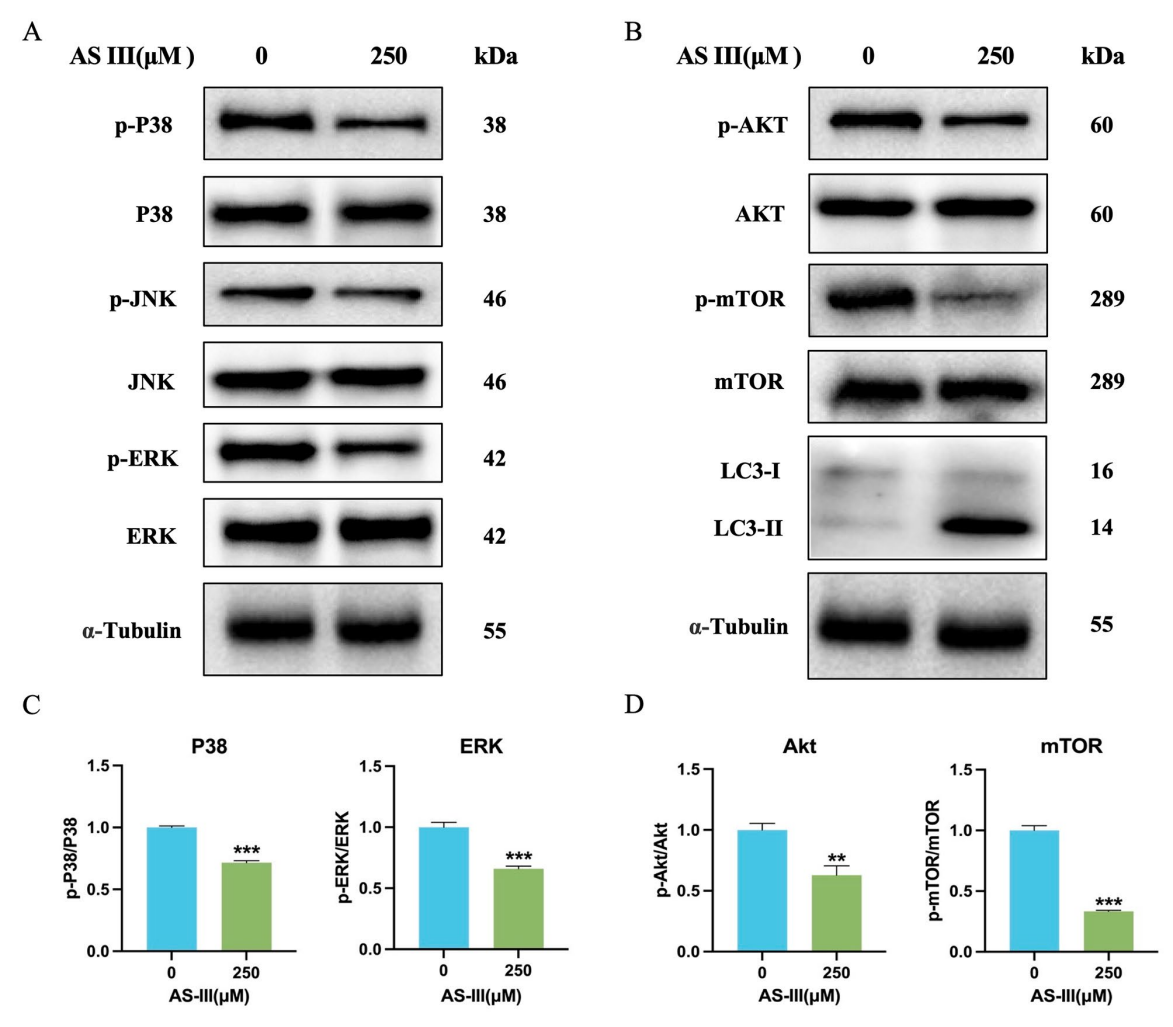
Effect of AS III on inhibition of the MAPK and AKT/mTOR signaling pathway. (**A-B**) Expression of the MAPKs and AKT/mTOR signaling pathway proteins in the presence and absence of AS III (250µM). (**C**) The ratios of p-P38/P38 and p-ERK/ERK were quantified from the Western blot data in (**A**). (**D**) The ratios of p-AKT/AKT and p-mTOR/mTOR were quantified from the western blot data in (**B**). Values are expressed as mean ± S.E.M. *P < 0.05, **P < 0.01, ***P < 0.001, compared untreated (0µM AS III) group

To characterize the downstream signaling pathways that mediate AS III-induced apoptosis, we evaluated the levels of key proteins involved in MAPK signaling [33]. AS III significantly reduced the phosphorylation of MAPKs (Fig. 8A), including extracellular signal-regulated kinase (ERK) and P38 mitogen-activated protein kinase (P38) (*P* < 0.01), but not c-Jun N-terminal kinase (JNK) (Fig. 8C).

We also determined whether the AKT/mTOR signaling pathway may be involved in AS III-induced autophagy in A549 cells (Fig. 8B). The results demonstrate that AS III significantly decreases the phosphorylation of both AKT and mTOR (*P* < 0.01) (Fig. 8D). Collectively, these results suggest that AS III modulates both the MAPK (ERK and P38) and AKT/mTOR pathways.

## Discussion

Despite coordinated efforts to develop effective therapies, lung cancer remains one of the most common causes of cancer-related deaths [34, 35]. Astragaloside analogues have been shown to have antitumor effects against a variety of cancers; therefore, we aimed to explore the potential efficacy and mechanisms of AS III in NSCLC cells by evaluating target proteins. Our experiments demonstrated that AS III inhibits the viability of lung cancer cells in a concentration-dependent manner. Thus, we used a combination of quantitative proteomics and western blotting to explore the mechanism of action of AS III in inhibiting the proliferation of A549 cells. Quantitative proteomics results demonstrated that AS III alters the protein expression levels of A549 cells, and 258 DEPs were identified, of which 198 were down-regulated and 60 were upregulated. By further analyzing their GO functional enrichment and KEGG pathways [36–38], we determined that the biological process term “Positive regulation of apoptotic process” was most highly enriched, suggesting that AS III may inhibit cell proliferation by inducing apoptosis in A549 cells.

Among the proteins identified by proteomic analysis, ANXA1 had a high connectivity score, suggesting a key role in mediating AS III activity. ANXA1 is the first characterized member of the annexin superfamily [39], which is composed of calcium-dependent phospholipid-binding proteins that are involved in physiological processes such as differentiation, apoptosis, proliferation and inflammation [40]. Resveratrol has been shown to significantly increase the expression levels of ANXA1 and Caspase-3 and inhibit the expression levels of Bcl-2 in human prokaryotic cells (HL-60) in a time-dependent manner. Furthermore, evidence suggest that ANXA1 may act similarly to Caspase-3 in inducing apoptosis [41]. In the present study, the expression of ANXA1 was significantly reduced in A549 cells after AS III treatment, which is consistent with the possibility that ANXA1 may be involved in AS III-induced apoptosis.

Caspase 3 is an important component of the apoptotic process [42], its activation and overexpression can directly induce apoptosis [43], Caspase 3 promotes apoptosis by degrading its enzyme substrate, PARP 1, which is known as the “death substrate“ [44, 45]. Under normal conditions, Caspase 3 is inactive in the cytoplasm and exists as the Pro-Caspase 3 form [46]. When cells are stimulated to undergo apoptosis, however, Pro-Caspase 3 is degraded to the active form, Cleaved-Caspase 3 [47] and PARP 1 is subsequently degraded to Cleaved-PARP 1, which leads to apoptosis [48–50]. In the present study, the expression of Caspase 3, PARP 1 and their cleaved counterparts were detected in A549 cells after AS III treatment, The results provide further evidence that AS III inhibits the viability of A549 cells by inducing apoptosis.

MAPKs, including P38, JNK, and ERK, are mediators of classical pathways involved in stress responses, including apoptosis. Studies have shown that the ERK signaling pathway is involved in cell growth, development and differentiation and is a key signaling pathway for apoptosis. ERK, as mediator of the MAPK/ERK signaling pathway [51], transduces signals from surface receptors to the nucleus [52], ERK is directly involved in the activation of a large number of downstream substrates, and its overactivation accelerates the differentiation process and promotes the proliferation of tumor cells [53]. P38 regulates the inflammatory response and can be activated by ERK phosphorylation [54], which further induces transcription factors to promote apoptosis and inhibit cell proliferation. JNK, also known as stress-activated protein kinase [55], has a similar ability to promote apoptosis when activated for an extended period [56, 57]. In this study, AS III was demonstrated to activate ERK and P38, but not JNK, which could potentially be explained by the short (24 h) time course or the inherent redundancy in these pathways. However, it is possible that dynamic studies would demonstrate a role for JNK activation at different times of AS III administration.

As an additional potential mechanism, we evaluated the effects of AS III on the AKT/mTOR pathway, which has been shown to induce cell proliferation, autophagy, and apoptosis in a variety of diseases [58]. Our results demonstrate that AS III leads to reduced phosphorylation of both AKT and mTOR and activation of Beclin-1. When autophagy is formed, cytoplasmic LC3-I enzymatically cleaves a small segment of polypeptide to convert it into membrane LC3-II, and as the concentration of AS III increases, the ratio of LC3-I/II decreases, which indicates that autophagy is at a high level. Changes in P62 expression is commonly used to monitor the autophagic flow, which is a bridge between LC3 and ubiquitinated proteins to be degraded [59], and the blockage of fusion of autophagosomes with lysosomes in the late stage of autophagy leads to a significant accumulation of P62 [60]. Elevated P62 levels accompanied by autophagy activation are usually associated with toxic stimuli and oxidative stress, therefore, we determine changes in the activity of cellular autophagy by focusing on the dynamics of autophagic flow, i.e., autophagosome formation, fusion of autophagosomes with lysosomes, and substrate degradation. Our experiments provide an additional potential explanation for the effects of AS III in inducing apoptosis and autophagy of A549 cells.

The present study has some limitations. First, we investigated the mechanism of action of AS III through in vitro experiments. Second, we used a single NSCLC cell line for western blot validation. Additional experiments to evaluate protein changes in mouse models or in normal lung cells may better reveal the specificity of AS III action.

## Conclusion

Our results suggest that AS III treatment exerts a therapeutic effect on NSCLC by inducing apoptosis and autophagy. These results may be mediated by a variety of mechanisms, including the P38, ERK and AKT/mTOR signaling pathways.

### Electronic supplementary material

Below is the link to the electronic supplementary material.

Supplementary Material 1

Supplementary Material 2

Supplementary Material 3

## Data Availability

All data supporting the article is provided in this article.

## Abbreviations

AR: Astragali Radix
AS-III: Astragaloside III
BP: Biological Process
CC: Cellular Component
CCK-8: Cell Counting Kit-8
DDP: Cisplatin
DEPs: Differentially expressed proteins
DMSO: Dimethyl sulfoxide
ERK: Extracellular signal-regulated kinase
GO: Gene Ontology
FBS: Fetal bovine serum
FDR: False discovery rate
JNK: c-Jun N-terminal kinase
JWBSYQF: Jia-Wei-Bu-Shen-Yi-Qi formula
KEGG: Kyoto Encyclopedia of Genes and Genomes
MAPK: Mitogen activated protein kinase
MF: Molecular Function
NSCLC: Non-small cell lung cancer
PARP: Poly-ADP-ribose polymerase
PPI: Protein-protein interaction
PVDF: Polyvinylidene difluoride
TMT: Tandem Mass Tag
