# Supplementary material for: Proteomic analysis reveals the molecular mechanism of Astragaloside in the treatment of non-small cell lung cancer by inducing apoptosis
Source: BMC Complement Med Ther. 2023 Dec 15;23:461. doi: 10.1186/s12906-023-04305-0 (PMC10722856; doi:10.1186/s12906-023-04305-0)
Supplement: Supplementary file 1 — Supplementary Material 1 [file 12906_2023_4305_MOESM1_ESM.doc]

Table 1. List of up-regulated proteins in AS-III-treated A549 cells

| Accession | Gene Name | Description | P-value | FC |
| --- | --- | --- | --- | --- |
| P02749 | APOH | Beta-2-glycoprotein 1 | 0.000413 | 4.232178 |
| O95800 | GPR75 | Probable G-protein coupled receptor 75 | 0.002187 | 4.032075 |
| O00767 | SCD | Stearoyl-CoA desaturase | 5.11E-07 | 3.932182 |
| A4FU01 | MTMR11 | Myotubularin-related protein 11 | 7.46E-05 | 3.756243 |
| O95864 | FADS2 | Acyl-CoA 6-desaturase | 2.64E-06 | 3.602417 |
| P37268 | FDFT1 | Squalene synthase | 1.29E-07 | 3.476777 |
| Q15800 | MSMO1 | Methylsterol monooxygenase 1 | 3.83E-07 | 3.357298 |
| Q9GZP1 | NRSN2 | Neurensin-2 | 1.32E-09 | 3.345464 |
| O00220 | TNFRSF10A | Tumor necrosis factor receptor superfamily member 10A | 8.03E-05 | 3.338937 |
| Q96QD8 | SLC38A2 | Sodium-coupled neutral amino acid transporter 2 | 7.39E-05 | 3.333694 |
| Q9NV92 | NDFIP2 | NEDD4 family-interacting protein 2 | 4.7E-07 | 3.270689 |
| Q9NQ39 | RPS10P5 | Putative 40S ribosomal protein S10-like | 5.55E-05 | 3.253057 |
| O60637 | TSPAN3 | Tetraspanin-3 | 3.81E-09 | 3.217185 |
| A0A1W2PR48 | TLE7 | Transducin-like enhancer protein 7 | 0.000128 | 3.204414 |
| P38571 | LIPA | Lysosomal acid lipase/cholesteryl ester hydrolase | 1.73E-05 | 2.905762 |
| Q13772 | NCOA4 | Nuclear receptor coactivator 4 | 0.00013 | 2.846154 |
| P41273 | TNFSF9 | Tumor necrosis factor ligand superfamily member 9 | 0.000153 | 2.829584 |
| Q13501 | SQSTM1 | Sequestosome-1 | 2.45E-07 | 2.813155 |
| P01042 | KNG1 | Kininogen-1 | 0.00349 | 2.791943 |
| Q9BZM5 | ULBP2 | UL16-binding protein 2 | 0.00117 | 2.780718 |
| Q14534 | SQLE | Squalene monooxygenase | 3.42E-07 | 2.761166 |
| P14210 | HGF | Hepatocyte growth factor | 0.000298 | 2.71402 |
| Q9NZV1 | CRIM1 | Cysteine-rich motor neuron 1 protein | 7.11E-08 | 2.712297 |
| Q16850 | CYP51A1 | Lanosterol 14-alpha demethylase | 5.81E-08 | 2.710111 |
| P29279 | CCN2 | CCN family member 2 | 7.45E-08 | 2.684938 |
| P01130 | LDLR | Low-density lipoprotein receptor | 2.08E-07 | 2.661785 |
| P04114 | APOB | Apolipoprotein B-100 | 1.49E-06 | 2.563474 |
| Q9Y287 | ITM2B | Integral membrane protein 2B | 4.59E-07 | 2.544528 |
| P10451 | SPP1 | Osteopontin | 1.36E-07 | 2.503285 |
| P08962 | CD63 | CD63 antigen | 7.97E-06 | 2.481723 |
| O43291 | SPINT2 | Kunitz-type protease inhibitor 2 | 1.39E-05 | 2.461705 |
| P02787 | TF | Serotransferrin | 2.34E-06 | 2.410486 |
| Q13145 | BAMBI | BMP and activin membrane-bound inhibitor homolog | 0.000132 | 2.406729 |
| Q8NHS3 | MFSD8 | Major facilitator superfamily domain-containing protein 8 | 6.82E-05 | 2.308106 |
| O94925 | GLS | Glutaminase kidney isoform, mitochondrial | 4.81E-05 | 2.290827 |
| Q99650 | OSMR | Oncostatin-M-specific receptor subunit beta | 7.63E-08 | 2.279623 |
| P02788 | LTF | Lactotransferrin | 1.23E-06 | 2.237556 |
| Q9C0H2 | TTYH3 | Protein tweety homolog 3 | 1.37E-05 | 2.201281 |
| P53801 | PTTG1IP | Pituitary tumor-transforming gene 1 protein-interacting protein | 4.24E-06 | 2.17965 |
| Q9H2H9 | SLC38A1 | Sodium-coupled neutral amino acid transporter 1 | 5.88E-05 | 2.178784 |
| Q9H3H1 | TRIT1 | tRNA dimethylallyltransferase | 0.000514 | 2.175466 |
| Q9Y320 | TMX2 | Thioredoxin-related transmembrane protein 2 | 2.48E-05 | 2.171689 |
| O95150 | TNFSF15 | Tumor necrosis factor ligand superfamily member 15 | 0.001784 | 2.170828 |
| O00555 | CACNA1A | Voltage-dependent P/Q-type calcium channel subunit alpha-1A | 6.71E-05 | 2.145891 |
| Q99732 | LITAF | Lipopolysaccharide-induced tumor necrosis factor-alpha factor OS=Homo sapiens | 0.000249 | 2.130282 |
| P78504 | JAG1 | Protein jagged-1 | 1.24E-06 | 2.09119 |
| P09601 | HMOX1 | Heme oxygenase 1 | 7.96E-05 | 2.089996 |
| Q8TEY7 | USP33 | Ubiquitin carboxyl-terminal hydrolase 33 | 9.1E-05 | 2.088803 |
| O43657 | TSPAN6 | Tetraspanin-6 | 1.01E-07 | 2.078492 |
| P48449 | LSS | Lanosterol synthase | 8.88E-08 | 2.067868 |
| P15514 | AREG | Amphiregulin | 8.41E-06 | 2.065926 |
| O43934 | MFSD11 | UNC93-like protein MFSD11 | 1.77E-06 | 2.051106 |
| P37173 | TGFBR2 | TGF-beta receptor type-2 | 8.66E-08 | 2.042982 |
| O60353 | FZD6 | Frizzled-6 | 4.9E-07 | 2.024575 |
| Q9UGQ3 | SLC2A6 | Solute carrier family 2, facilitated glucose transporter member 6 | 2.62E-05 | 2.024197 |
| Q15043 | SLC39A14 | Metal cation symporter ZIP14 | 3.49E-06 | 2.023432 |
| P13224 | GP1BB | Platelet glycoprotein Ib beta chain | 0.00259 | 2.016591 |
| O75616 | ERAL1 | GTPase Era, mitochondrial | 2.42E-05 | 2.014318 |
| Q9UPY5 | SLC7A11 | Cystine/glutamate transporter | 1.85E-05 | 2.011291 |
| P04062 | GBA | Lysosomal acid glucosylceramidase | 9.75E-05 | 2.004131 |

Table 2. List of down-regulated proteins in AS-III-treated A549 cells

| Accession | Gene Name | Description | P-value | FC |
| --- | --- | --- | --- | --- |
| P50238 | CRIP1 | Cysteine-rich protein 1 | 0.000157 | 0.498876 |
| O75884 | RBBP9 | Serine hydrolase RBBP9 | 2.18E-05 | 0.498876 |
| P00441 | SOD1 | Superoxide dismutase [Cu-Zn] | 1.51E-06 | 0.498689 |
| Q7Z3E5 | ARMC9 | LisH domain-containing protein ARMC9 | 9.42E-05 | 0.498408 |
| P27348 | YWHAQ | 14-3-3 protein theta OS=Homo sapiens | 1.17E-05 | 0.49794 |
| Q15796 | SMAD2 | Mothers against decapentaplegic homolog 2 | 7.86E-06 | 0.49794 |
| Q15435 | PPP1R7 | Protein phosphatase 1 regulatory subunit 7 | 7.99E-06 | 0.497286 |
| Q8TEA8 | DTD1 | D-aminoacyl-tRNA deacylase 1 | 8.21E-06 | 0.497006 |
| Q9ULZ3 | PYCARD | Apoptosis-associated speck-like protein containing a CARD | 3.13E-05 | 0.496819 |
| Q9HC38 | GLOD4 | Glyoxalase domain-containing protein 4 | 1.22E-05 | 0.496726 |
| Q8N4P3 | HDDC3 | Guanosine-3',5'-bis(diphosphate) 3'-pyrophosphohydrolase MESH1 | 7.9E-05 | 0.495607 |
| P31946 | YWHAB | 14-3-3 protein beta/alpha | 3.74E-06 | 0.494395 |
| Q15181 | PPA1 | Inorganic pyrophosphatase | 1.8E-05 | 0.49421 |
| Q9HB71 | CACYBP | Calcyclin-binding protein | 0.000666 | 0.493652 |
| P07195 | LDHB | L-lactate dehydrogenase B chain | 0.000115 | 0.493094 |
| Q86VP6 | CAND1 | Cullin-associated NEDD8-dissociated protein 1 | 1.07E-05 | 0.492816 |
| P35237 | SERPINB6 | Serpin B6 | 6.77E-05 | 0.492445 |
| Q9UHY7 | ENOPH1 | Enolase-phosphatase | 1.48E-05 | 0.492259 |
| Q9HCJ3 | RAVER2 | Ribonucleoprotein PTB-binding 2 | 0.003275 | 0.492259 |
| P46527 | CDKN1B | Cyclin-dependent kinase inhibitor 1B | 0.002956 | 0.492167 |
| P41236 | PPP1R2 | Protein phosphatase inhibitor 2 | 1.76E-05 | 0.491424 |
| Q14240 | EIF4A2 | Eukaryotic initiation factor 4A-II | 9.92E-05 | 0.490591 |
| O15305 | PMM2 | Phosphomannomutase 2 | 4.8E-05 | 0.490404 |
| Q8NFI3 | ENGASE | Cytosolic endo-beta-N-acetylglucosaminidase | 0.006232 | 0.490404 |
| Q00688 | FKBP3 | Peptidyl-prolyl cis-trans isomerase FKBP3 | 3.09E-06 | 0.490313 |
| Q9UKY7 | CDV3 | Protein CDV3 homolog | 1.33E-05 | 0.48874 |
| P52565 | ARHGDIA | Rho GDP-dissociation inhibitor 1 | 0.000137 | 0.488554 |
| P62266 | RPS23 | 40S ribosomal protein S23 | 1.02E-05 | 0.488281 |
| Q3ZCM7 | TUBB8 | Tubulin beta-8 chain | 0.000582 | 0.488095 |
| Q9NR45 | NANS | Sialic acid synthase | 3.45E-05 | 0.488095 |
| P30419 | NMT1 | Glycylpeptide N-tetradecanoyltransferase 1 | 7.68E-06 | 0.487909 |
| O95336 | PGLS | 6-phosphogluconolactonase | 3.23E-06 | 0.487818 |
| Q96TA1 | NIBAN2 | Protein Niban 2 | 0.000125 | 0.487265 |
| Q96GG9 | DCUN1D1 | DCN1-like protein 1 | 1.96E-05 | 0.487079 |
| Q9BT73 | PSMG3 | Proteasome assembly chaperone 3 | 6.74E-05 | 0.487079 |
| Q9BW30 | TPPP3 | Tubulin polymerization-promoting protein family member 3 | 0.013081 | 0.486989 |
| P16083 | NQO2 | Ribosyldihydronicotinamide dehydrogenase [quinone] | 8.41E-06 | 0.486713 |
| Q9NPF4 | OSGEP | Probable tRNA N6-adenosine threonylcarbamoyltransferase 6 | 0.000299 | 0.48616 |
| P58546 | MTPN | Myotrophin | 6.67E-05 | 0.48607 |
| P22314 | UBA1 | Ubiquitin-like modifier-activating enzyme 1 | 7.39E-05 | 0.484781 |
| Q96K17 | BTF3L4 | Transcription factor BTF3 homolog 4 | 1.17E-05 | 0.484045 |
| P61081 | UBE2M | NEDD8-conjugating enzyme Ubc12 | 1.82E-05 | 0.48368 |
| P15428 | HPGD | 15-hydroxyprostaglandin dehydrogenase [NAD(+)] | 0.000731 | 0.482854 |
| Q86WR0 | CCDC25 | Coiled-coil domain-containing protein 25 | 9.09E-06 | 0.482854 |
| Q96G03 | PGM2 | Phosphoglucomutase-2 | 2.13E-05 | 0.482305 |
| P35754 | GLRX | Glutaredoxin-1 | 0.000314 | 0.481392 |
| P52209 | PGD | 6-phosphogluconate dehydrogenase, decarboxylating | 5.1E-05 | 0.481111 |
| P26447 | S100A4 | Protein S100-A4 | 0.000385 | 0.480385 |
| P00352 | ALDH1A1 | Aldehyde dehydrogenase 1A1 | 3.75E-05 | 0.479748 |
| Q8NC51 | SERBP1 | Plasminogen activator inhibitor 1 RNA-binding protein | 2.33E-05 | 0.478743 |
| P61970 | NUTF2 | Nuclear transport factor 2 | 3.23E-06 | 0.478655 |
| Q7Z745 | MROH2B | Maestro heat-like repeat-containing protein family member 2B | 0.000214 | 0.477105 |
| P49354 | FNTA | Protein farnesyltransferase/geranylgeranyltransferase type-1 subunit alpha | 2.86E-05 | 0.476015 |
| O95749 | GGPS1 | Geranylgeranyl pyrophosphate synthase | 1.94E-05 | 0.475558 |
| P09525 | ANXA4 | Annexin A4 | 0.000548 | 0.47547 |
| P00338 | LDHA | L-lactate dehydrogenase A chain | 2.51E-05 | 0.474654 |
| P27816 | MAP4 | Microtubule-associated protein 4 | 3.69E-05 | 0.47447 |
| P49915 | GMPS | GMP synthase [glutamine-hydrolyzing] | 2.17E-05 | 0.474383 |
| Q9NTK5 | OLA1 | Obg-like ATPase 1 | 4.95E-05 | 0.474383 |
| Q9BRA2 | TXNDC17 | Thioredoxin domain-containing protein 17 | 8.59E-06 | 0.473568 |
| Q9UL46 | PSME2 | Proteasome activator complex subunit 2 | 1.57E-05 | 0.473481 |
| O95834 | EML2 | Echinoderm microtubule-associated protein-like 2 | 0.000374 | 0.473297 |
| Q04760 | GLO1 | Lactoylglutathione lyase | 1.97E-05 | 0.473209 |
| P61088 | UBE2N | Ubiquitin-conjugating enzyme E2 N | 2.77E-06 | 0.473025 |
| P31949 | S100A11 | Protein S100-A11 | 9.14E-06 | 0.470772 |
| Q9BTT0 | ANP32E | Acidic leucine-rich nuclear phosphoprotein 32 family member E | 5.74E-05 | 0.470491 |
| Q9C0B1 | FTO | Alpha-ketoglutarate-dependent dioxygenase FTO | 0.000114 | 0.469508 |
| P20962 | PTMS | Parathymosin | 0.000699 | 0.468784 |
| O75368 | SH3BGRL | SH3 domain-binding glutamic acid-rich-like protein | 0.000167 | 0.468073 |
| P27707 | DCK | Deoxycytidine kinase | 3.38E-05 | 0.467976 |
| Q9HC35 | EML4 | Echinoderm microtubule-associated protein-like 4 | 2.97E-05 | 0.467804 |
| Q99584 | S100A13 | Protein S100-A13 | 1.22E-05 | 0.467621 |
| O75391 | SPAG7 | Sperm-associated antigen 7 | 3.28E-05 | 0.467351 |
| Q53FT3 | HIKESHI | Protein Hikeshi | 6.07E-06 | 0.467266 |
| O75223 | GGCT | Gamma-glutamylcyclotransferase | 2.05E-05 | 0.466544 |
| P30041 | PRDX6 | Peroxiredoxin-6 | 4.73E-05 | 0.466007 |
| Q9BXV9 | GON7 | EKC/KEOPS complex subunit GON7 | 1.91E-05 | 0.465385 |
| Q9Y570 | PPME1 | Protein phosphatase methylesterase 1 | 1.03E-05 | 0.464933 |
| Q13442 | PDAP1 | 28 kDa heat- and acid-stable phosphoprotein | 8.99E-06 | 0.463861 |
| P68036 | UBE2L3 | Ubiquitin-conjugating enzyme E2 L3 | 4.52E-06 | 0.463776 |
| P23743 | DGKA | Diacylglycerol kinase alpha | 0.000154 | 0.463593 |
| Q2TAA2 | IAH1 | Isoamyl acetate-hydrolyzing esterase 1 homolog | 8.61E-05 | 0.463142 |
| P16930 | FAH | Fumarylacetoacetase | 8.41E-06 | 0.46296 |
| P06132 | UROD | Uroporphyrinogen decarboxylase | 0.0002 | 0.461988 |
| Q14019 | COTL1 | Coactosin-like protein | 0.000115 | 0.461103 |
| P40261 | NNMT | Nicotinamide N-methyltransferase | 0.000108 | 0.461005 |
| Q06323 | PSME1 | Proteasome activator complex subunit 1 | 6.82E-05 | 0.460654 |
| P09211 | GSTP1 | Glutathione S-transferase P | 2.8E-05 | 0.460387 |
| P37840 | SNCA | Alpha-synuclein | 0.002688 | 0.459938 |
| P53004 | BLVRA | Biliverdin reductase A | 2.72E-05 | 0.459854 |
| P00813 | ADA | Adenosine deaminase | 1.21E-05 | 0.459504 |
| P42330 | AKR1C3 | Aldo-keto reductase family 1 member C3 | 2.95E-05 | 0.459139 |
| P55263 | ADK | Adenosine kinase | 0.000142 | 0.458789 |
| Q9Y2V2 | CARHSP1 | Calcium-regulated heat-stable protein 1 | 5.48E-05 | 0.458341 |
| P39748 | FEN1 | Flap endonuclease 1 | 1.73E-05 | 0.457278 |
| P63241 | EIF5A | Eukaryotic translation initiation factor 5A-1 | 1.65E-05 | 0.457112 |
| P63104 | YWHAZ | 14-3-3 protein zeta/delta | 3.82E-06 | 0.456664 |
| P36871 | PGM1 | Phosphoglucomutase-1 | 2.71E-05 | 0.455869 |
| Q58FF6 | HSP90AB4P | Putative heat shock protein HSP 90-beta 4 | 0.048604 | 0.453852 |
| Q9NPH2 | ISYNA1 | Inositol-3-phosphate synthase 1 | 0.000193 | 0.453571 |
| Q9NZN4 | EHD2 | EH domain-containing protein 2 | 3.92E-05 | 0.453488 |
| P55327 | TPD52 | Tumor protein D52 | 3.89E-05 | 0.452169 |
| O60218 | AKR1B10 | Aldo-keto reductase family 1 member B10 | 2.11E-05 | 0.451906 |
| P37802 | TAGLN2 | Transgelin-2 | 0.000184 | 0.451642 |
| Q9Y237 | PIN4 | Peptidyl-prolyl cis-trans isomerase NIMA-interacting 4 | 6.37E-07 | 0.451642 |
| O95243 | MBD4 | Methyl-CpG-binding domain protein 4 | 0.027463 | 0.450852 |
| Q969M7 | UBE2F | NEDD8-conjugating enzyme UBE2F | 0.000621 | 0.450852 |
| O14559 | ARHGAP33 | Rho GTPase-activating protein 33 | 3.39E-06 | 0.450508 |
| O60869 | EDF1 | Endothelial differentiation-related factor 1 | 6.41E-06 | 0.450063 |
| P06744 | GPI | Glucose-6-phosphate isomerase | 1.38E-05 | 0.449457 |
| P08397 | HMBS | Porphobilinogen deaminase | 1.5E-05 | 0.449457 |
| Q7L9L4 | MOB1B | MOB kinase activator 1B | 2.92E-05 | 0.449357 |
| Q96AT1 | KIAA1143 | Uncharacterized protein KIAA1143 | 5.97E-05 | 0.449357 |
| Q9H3Q1 | CDC42EP4 | Cdc42 effector protein 4 | 0.000266 | 0.449194 |
| P23381 | WARS1 | Tryptophan--tRNA ligase, cytoplasmic | 4.47E-06 | 0.44875 |
| Q9NWV4 | CZIB | CXXC motif containing zinc binding protein | 3.2E-05 | 0.447178 |
| O60664 | PLIN3 | Perilipin-3 | 4.36E-06 | 0.446916 |
| Q96LR5 | UBE2E2 | Ubiquitin-conjugating enzyme E2 E2 | 1.09E-05 | 0.446474 |
| Q01469 | FABP5 | Fatty acid-binding protein 5 | 3.22E-05 | 0.445951 |
| P40925 | MDH1 | Malate dehydrogenase, cytoplasmic | 6.31E-06 | 0.442561 |
| Q8WVY7 | UBLCP1 | Ubiquitin-like domain-containing CTD phosphatase 1 | 0.000125 | 0.441881 |
| P30740 | SERPINB1 | Leukocyte elastase inhibitor | 8.09E-05 | 0.441701 |
| P30086 | PEBP1 | Phosphatidylethanolamine-binding protein 1 | 9.03E-06 | 0.440403 |
| O95989 | NUDT3 | Diphosphoinositol polyphosphate phosphohydrolase 1 | 1.8E-05 | 0.440403 |
| Q16719 | KYNU | Kynureninase | 4.89E-05 | 0.440065 |
| P49321 | NASP | Nuclear autoantigenic sperm protein | 1.25E-05 | 0.437298 |
| Q9Y2S6 | TMA7 | Translation machinery-associated protein 7 | 1.17E-05 | 0.436782 |
| Q14914 | PTGR1 | Prostaglandin reductase 1 | 4.38E-05 | 0.436266 |
| P27695 | APEX1 | DNA-(apurinic or apyrimidinic site) endonuclease | 1.63E-05 | 0.43472 |
| P36969 | GPX4 | Phospholipid hydroperoxide glutathione peroxidase | 0.013261 | 0.433435 |
| O95613 | PCNT | Pericentrin | 0.005377 | 0.433178 |
| Q14195 | DPYSL3 | Dihydropyrimidinase-related protein 3 | 0.005928 | 0.432999 |
| P06733 | ENO1 | Alpha-enolase | 9.09E-06 | 0.431818 |
| P16949 | STMN1 | Stathmin | 1.38E-05 | 0.430538 |
| P20810 | CAST | Calpastatin | 0.00016 | 0.428827 |
| Q9UBT2 | UBA2 | SUMO-activating enzyme subunit 2 | 9.14E-05 | 0.427297 |
| P49903 | SEPHS1 | Selenide, water dikinase 1 | 4.42E-05 | 0.427119 |
| P30838 | ALDH3A1 | Aldehyde dehydrogenase, dimeric NADP-preferring | 0.000155 | 0.426203 |
| P18669 | PGAM1 | Phosphoglycerate mutase 1 | 3.36E-06 | 0.425187 |
| Q13765 | NACA | Nascent polypeptide-associated complex subunit alpha | 4.33E-05 | 0.424679 |
| P09936 | UCHL1 | Ubiquitin carboxyl-terminal hydrolase isozyme L1 | 6.93E-05 | 0.424501 |
| P43490 | NAMPT | Nicotinamide phosphoribosyltransferase | 0.002769 | 0.424426 |
| Q05639 | EEF1A2 | Elongation factor 1-alpha 2 | 0.000778 | 0.422475 |
| P53999 | SUB1 | Activated RNA polymerase II transcriptional coactivator p15 | 7.68E-05 | 0.421464 |
| P78417 | GSTO1 | Glutathione S-transferase omega-1 | 2.42E-06 | 0.41844 |
| Q6PUV4 | CPLX2 | Complexin-2 | 0.000182 | 0.418114 |
| P08758 | ANXA5 | Annexin A5 | 0.000156 | 0.416504 |
| O14737 | PDCD5 | Programmed cell death protein 5 | 3.48E-05 | 0.415826 |
| Q969T4 | UBE2E3 | Ubiquitin-conjugating enzyme E2 E3 | 2.28E-05 | 0.414677 |
| Q8IU85 | CAMK1D | Calcium/calmodulin-dependent protein kinase type 1D | 0.000146 | 0.414677 |
| P09104 | ENO2 | Gamma-enolase | 0.000154 | 0.414177 |
| Q96C86 | DCPS | m7GpppX diphosphatase | 5.11E-05 | 0.413927 |
| P06703 | S100A6 | Protein S100-A6 | 0.000441 | 0.413854 |
| Q96C90 | PPP1R14B | Protein phosphatase 1 regulatory subunit 14B | 0.00087 | 0.413677 |
| P12429 | ANXA3 | Annexin A3 | 0.000454 | 0.413604 |
| O00193 | SMAP | Small acidic protein | 7.85E-05 | 0.413178 |
| Q15714 | TSC22D1 | TSC22 domain family protein 1 | 3.5E-06 | 0.412855 |
| Q8NDH3 | NPEPL1 | Probable aminopeptidase NPEPL1 | 0.002295 | 0.412429 |
| P33764 | S100A3 | Protein S100-A3 | 0.003188 | 0.410365 |
| P00558 | PGK1 | Phosphoglycerate kinase 1 | 9.8E-06 | 0.407955 |
| P49441 | INPP1 | Inositol polyphosphate 1-phosphatase | 8.01E-05 | 0.407707 |
| P57076 | CFAP298 | Cilia- and flagella-associated protein 298 | 6.71E-05 | 0.404423 |
| Q5TFE4 | NT5DC1 | 5'-nucleotidase domain-containing protein 1 | 1.92E-06 | 0.399405 |
| P30046 | DDT | D-dopachrome decarboxylase | 1.93E-06 | 0.399405 |
| P25815 | S100P | Protein S100-P | 0.0001 | 0.399091 |
| Q9BS40 | LXN | Latexin | 9.81E-06 | 0.398043 |
| P62328 | TMSB4X | Thymosin beta-4 | 1.49E-05 | 0.395917 |
| P14174 | MIF | Macrophage migration inhibitory factor | 3.03E-06 | 0.395361 |
| P26583 | HMGB2 | High mobility group protein B2 | 6.91E-05 | 0.392273 |
| P04083 | ANXA1 | Annexin A1 | 1.2E-06 | 0.390821 |
| Q9UK76 | JPT1 | Jupiter microtubule associated homolog 1 | 0.000154 | 0.390647 |
| P09429 | HMGB1 | High mobility group protein B1 | 1.14E-05 | 0.388099 |
| O15347 | HMGB3 | High mobility group protein B3 | 3.28E-05 | 0.386722 |
| Q9H098 | FAM107B | Protein FAM107B | 1.19E-05 | 0.384389 |
| P09960 | LTA4H | Leukotriene A-4 hydrolase | 2.86E-05 | 0.384083 |
| O75347 | TBCA | Tubulin-specific chaperone A | 7.38E-06 | 0.384083 |
| Q9GZP8 | IMUP | Immortalization up-regulated protein | 6.04E-05 | 0.383365 |
| Q01105 | SET | Protein SET | 5.05E-05 | 0.383126 |
| P56211 | ARPP19 | cAMP-regulated phosphoprotein 19 | 5E-05 | 0.382648 |
| P20290 | BTF3 | Transcription factor BTF3 | 4.31E-05 | 0.380435 |
| Q8WW12 | PCNP | PEST proteolytic signal-containing nuclear protein OS=Homo sapiens | 2.44E-05 | 0.38009 |
| Q9UBE0 | SAE1 | SUMO-activating enzyme subunit 1 | 4.07E-05 | 0.370973 |
| P40121 | CAPG | Macrophage-capping protein | 0.001138 | 0.359157 |
| Q93045 | STMN2 | Stathmin-2 | 3.06E-06 | 0.352265 |
| P60174 | TPI1 | Triosephosphate isomerase | 8.58E-06 | 0.337402 |
| Q92688 | ANP32B | Acidic leucine-rich nuclear phosphoprotein 32 family member B | 8.36E-06 | 0.336005 |
| Q96A00 | PPP1R14A | Protein phosphatase 1 regulatory subunit 14A | 0.000343 | 0.334668 |
| P29401 | TKT | Transketolase | 2.31E-05 | 0.33178 |
| P63313 | TMSB10 | Thymosin beta-10 | 2.97E-06 | 0.330008 |
| P39687 | ANP32A | Acidic leucine-rich nuclear phosphoprotein 32 family member A | 6.41E-05 | 0.308686 |
| P62861 | FAU | 40S ribosomal protein S30 | 1.8E-05 | 0.307831 |
| Q9H1E3 | NUCKS1 | Nuclear ubiquitous casein and cyclin-dependent kinase substrate 1 | 0.000106 | 0.305696 |
| P51858 | HDGF | Hepatoma-derived growth factor | 0.00046 | 0.294241 |
| P80297 | MT1X | Metallothionein-1X | 0.012667 | 0.288867 |
| P37837 | TALDO1 | Transaldolase | 0.000248 | 0.263865 |
| P51965 | UBE2E1 | Ubiquitin-conjugating enzyme E2 E1 | 2.8E-05 | 0.250938 |
| P06454 | PTMA | Prothymosin alpha | 3.25E-05 | 0.206668 |
| Q9ULE4 | FAM184B | Protein FAM184B | 0.040122 | 0.0478 |

Table 3. List of differentially expressed proteins associated with apoptosis

| Accession | Gene Name | Description | P-value | | FC | |
| --- | --- | --- | --- | --- | --- | --- |
| O00220 | TNFRSF10A | Tumor necrosis factor receptor superfamily member 10A | 8.03E-05 | | 3.338937 | |
| Q13501 | SQSTM1 | Sequestosome-1 | 2.45E-07 | | 2.813155 | |
| P14210 | HGF | Hepatocyte growth factor | 0.000298 | | 2.71402 | |
| P02788 | LTF | Lactotransferrin | 1.23E-06 | | 2.237556 | |
| P09601 | HMOX1 | Heme oxygenase 1 | 7.96E-05 | | 2.089996 | |
| P63104 | YWHAZ | 14-3-3 protein zeta/delta | 3.82E-06 | | 0.456664 | |
| P00441 | SOD1 | Superoxide dismutase [Cu-Zn] | 1.51E-06 | | 0.498689 | |
| P37840 | SNCA | Alpha-synuclein | 0.002688 | | 0.459938 | |
| Q9ULZ3 | PYCARD | Apoptosis-associated speck-like protein containing a CARD | 3.13E-05 | | 0.496819 | |
| P06454 | PTMA | Prothymosin alpha | 3.25E-05 | | 0.206668 | |
| O14737 | PDCD5 | Programmed cell death protein 5 | 3.48E-05 | | 0.415826 | |
| P30419 | NMT1 | Glycylpeptide N-tetradecanoyltransferase 1 | 7.68E-06 | | 0.487909 | |
| Q96TA1 | NIBAN2 | Protein Niban 2 | 0.000125 | | 0.487265 | |
| P14174 | MIF | Macrophage migration inhibitory factor | 3.03E-06 | | 0.395361 | |
| P00338 | LDHA | L-lactate dehydrogenase A chain | 2.51E-05 | | 0.474654 | |
| P01042 | KNG1 | Kininogen-1 | 0.00349 | | 2.791943 | |
| P15428 | HPGD | 15-hydroxyprostaglandin dehydrogenase [NAD(+)] | 0.000731 | | 0.482854 | |
| P26583 | HMGB2 | High mobility group protein B2 | 6.91E-05 | | 0.392273 | |
| P09429 | HMGB1 | High mobility group protein B1 | 1.14E-05 | | 0.388099 | |
| P09211 | GSTP1 | Glutathione S-transferase P | 2.8E-05 | | 0.460387 | |
| Q04760 | GLO1 | Lactoylglutathione lyase | 1.97E-05 | | 0.473209 | |
| P06733 | ENO1 | Alpha-enolase | 9.09E-06 | | 0.431818 | |
| P63241 | EIF5A | Eukaryotic translation initiation factor 5A-1 | 1.65E-05 | | 0.457112 | |
| Q05639 | EEF1A2 | Elongation factor 1-alpha 2 | 0.000778 | | 0.422475 | |
| P50238 | CRIP1 | Cysteine-rich protein 1 | 0.000157 | | 0.498876 | |
| P46527 | CDKN1B | Cyclin-dependent kinase inhibitor 1B | 0.002956 | | 0.492167 | |
| Q8IU85 | CAMK1D | Calcium/calmodulin-dependent protein kinase type 1D | 0.000146 | | 0.414677 | |
| P52565 | ARHGDIA | Rho GDP-dissociation inhibitor 1 | 0.000137 | | 0.488554 | |
| P09525 | ANXA4 | Annexin A4 | | 0.000548 | | 0.47547 |
| P08758 | ANXA5 | Annexin A5 | | 0.000156 | | 0.416504 |
| P04083 | ANXA1 | Annexin A1 | | 1.2E-06 | | 0.390821 |
