# Supplementary figures and images for: Proteomic analysis reveals the molecular mechanism of Astragaloside in the treatment of non-small cell lung cancer by inducing apoptosis
Source: BMC Complement Med Ther. 2023 Dec 15;23:461. doi: 10.1186/s12906-023-04305-0 (PMC10722856; doi:10.1186/s12906-023-04305-0)

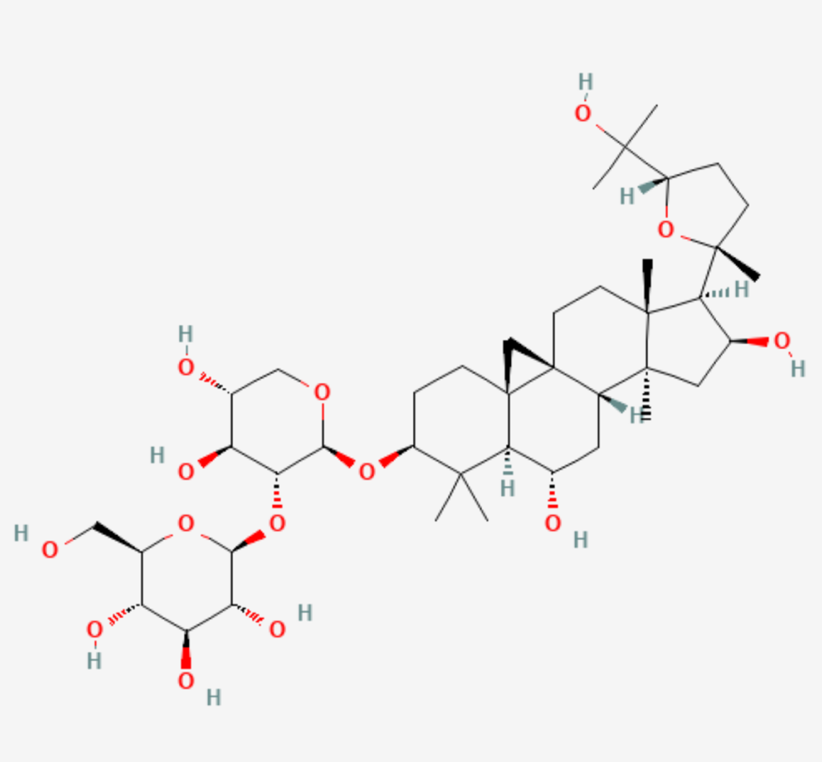


Fig.S1 Chemical structure of Astragaloside III

Supplement: Supplementary file 2 — Supplementary Material 2 [file 12906_2023_4305_MOESM2_ESM.doc]
