# Supplementary material for: Proteomic analysis reveals the molecular mechanism of Astragaloside in the treatment of non-small cell lung cancer by inducing apoptosis
Source: BMC Complement Med Ther. 2023 Dec 15;23:461. doi: 10.1186/s12906-023-04305-0 (PMC10722856; doi:10.1186/s12906-023-04305-0)
Supplement: Supplementary file 3 — Supplementary Material 3 [file 12906_2023_4305_MOESM3_ESM.pdf]

Western Blot Fig.5B

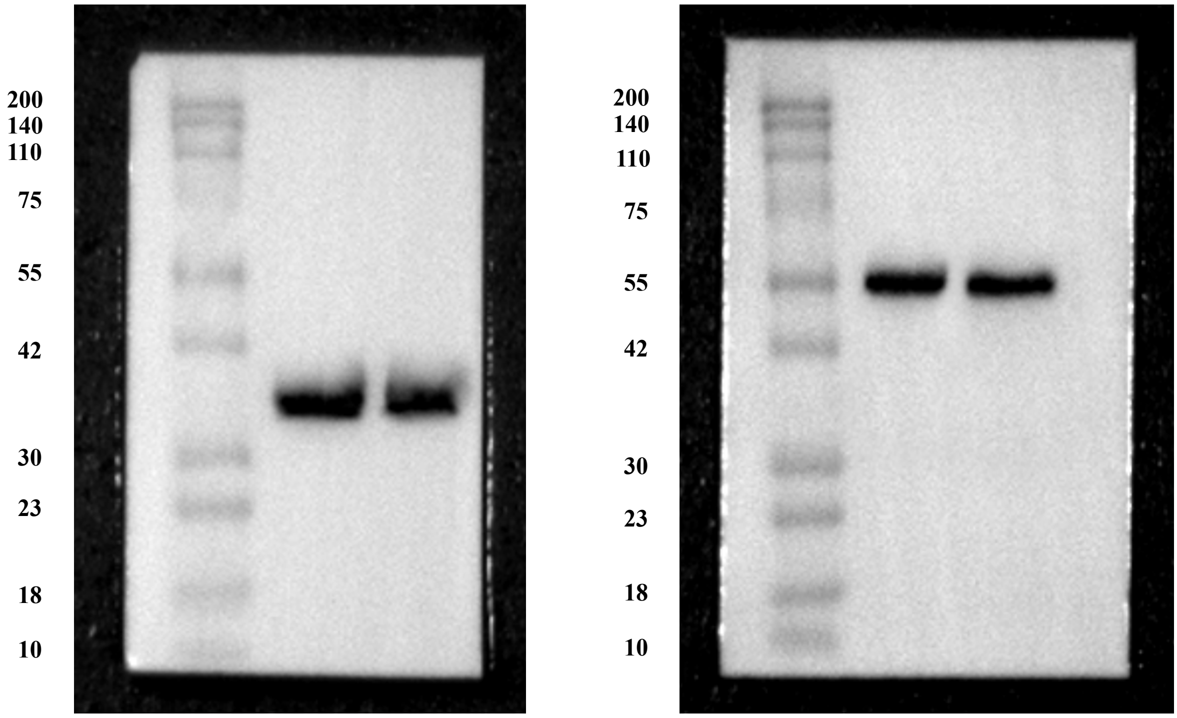

ANXA1 antibody

Tubulin antibody

Western Blot Fig.6C

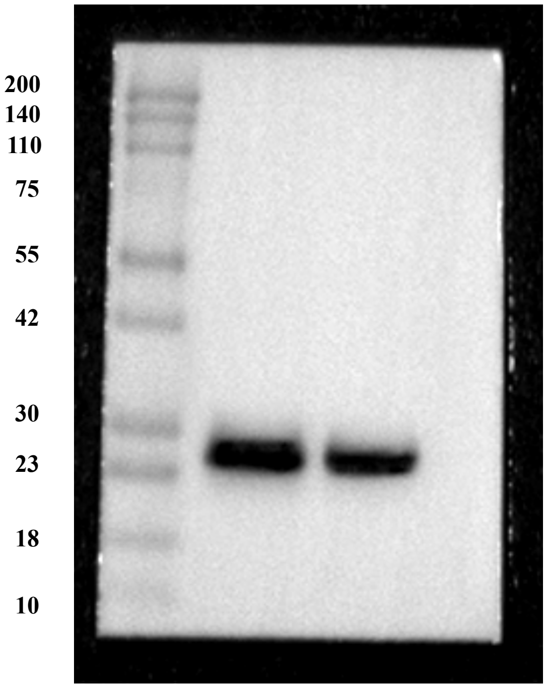

Bcl-2 antibody

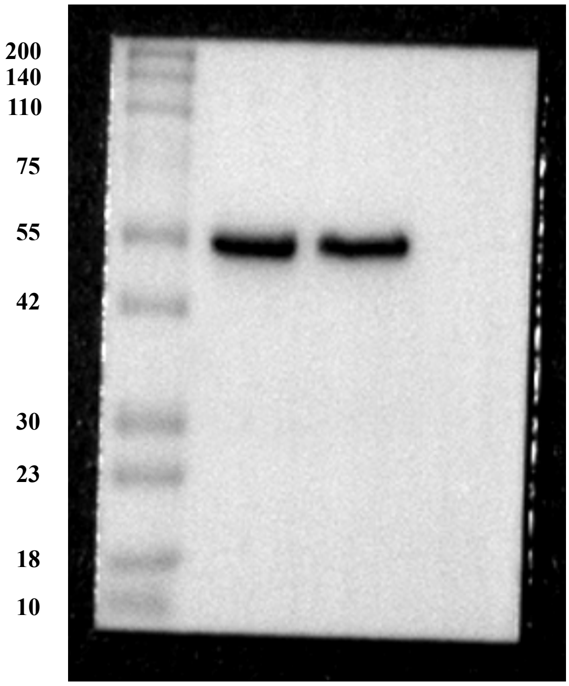

Tubulin antibody

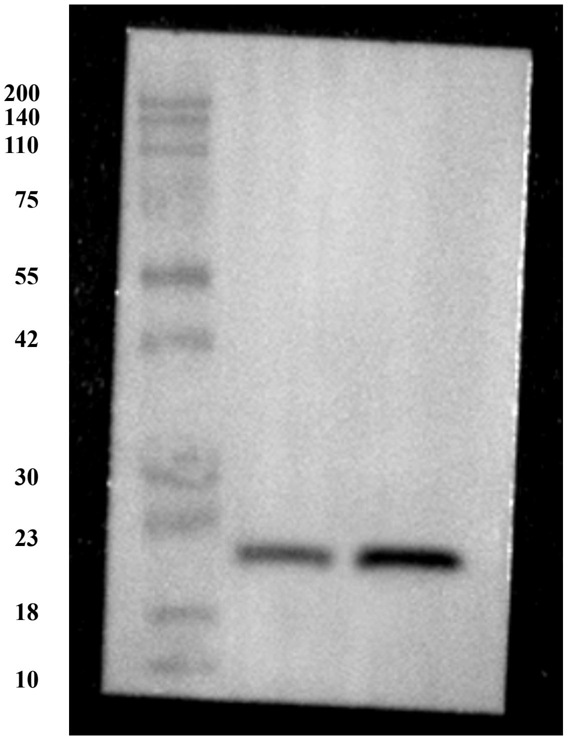

Bax antibody

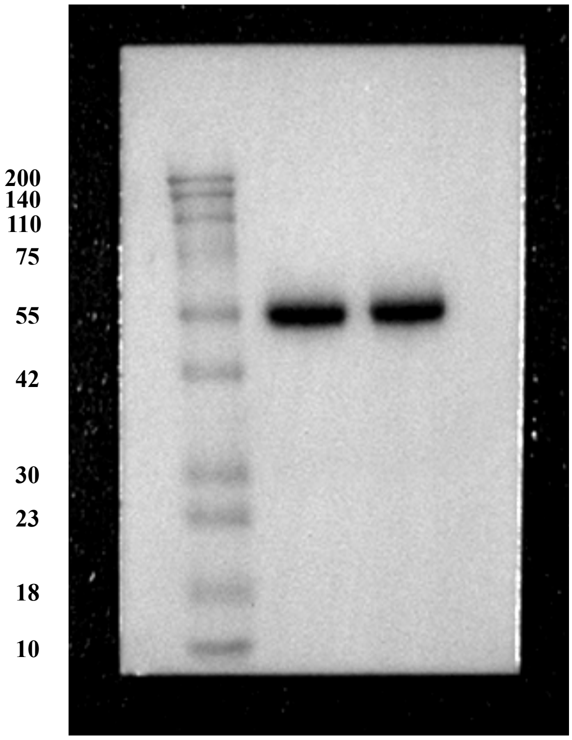

Tubulin antibody

Western Blot Fig.7A

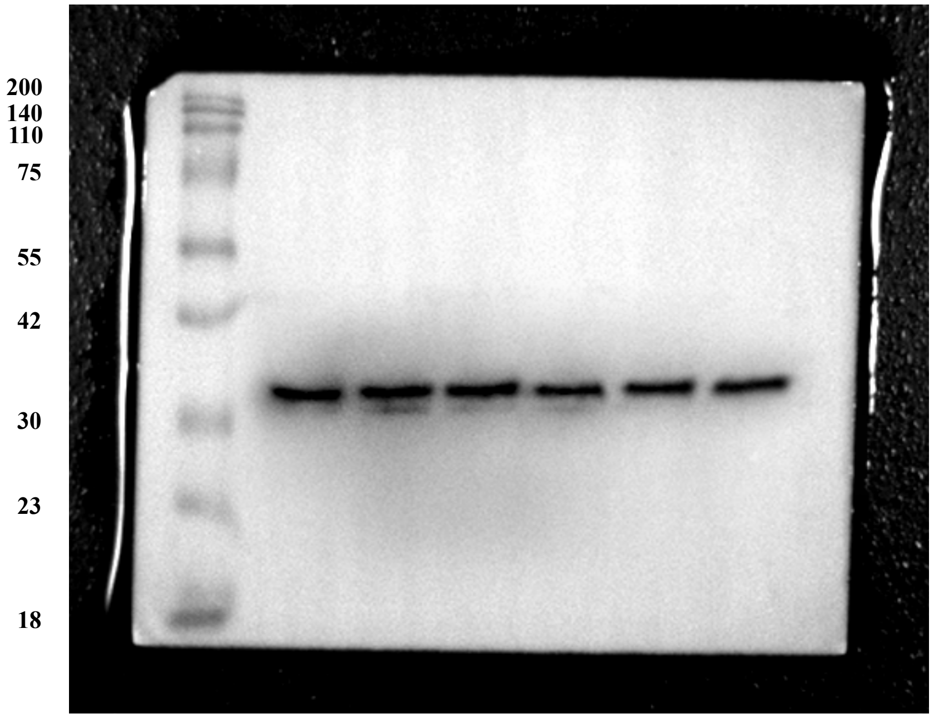

Caspase 3 antibody

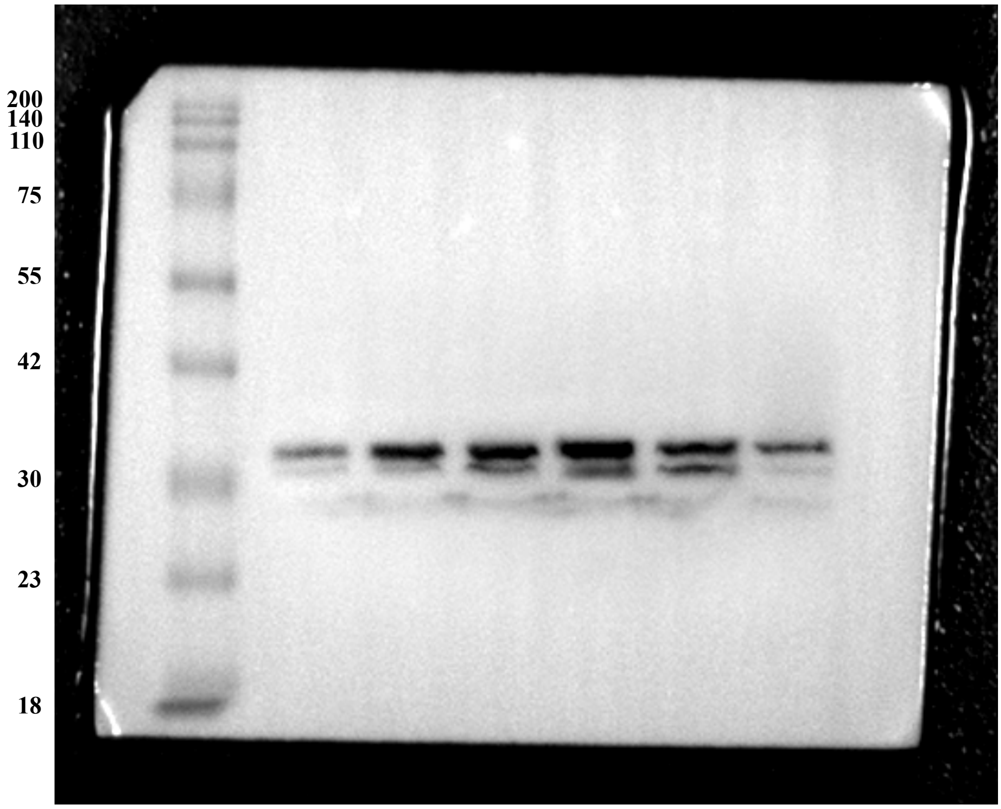

Cleaved-Caspase 3 antibody (1)

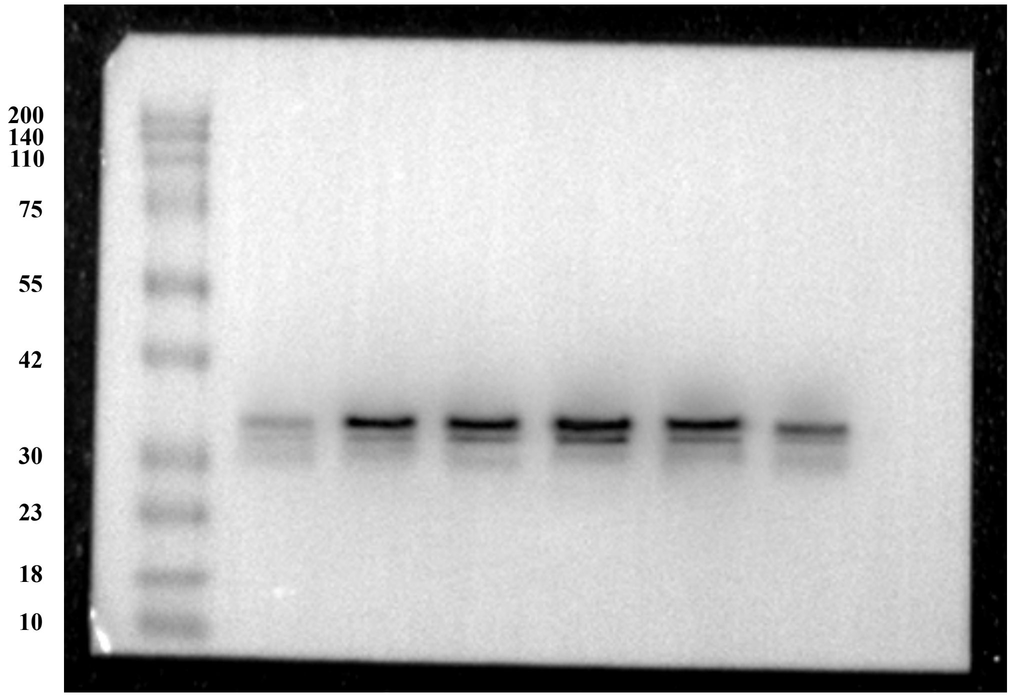

Cleaved-Caspase 3 antibody (2)

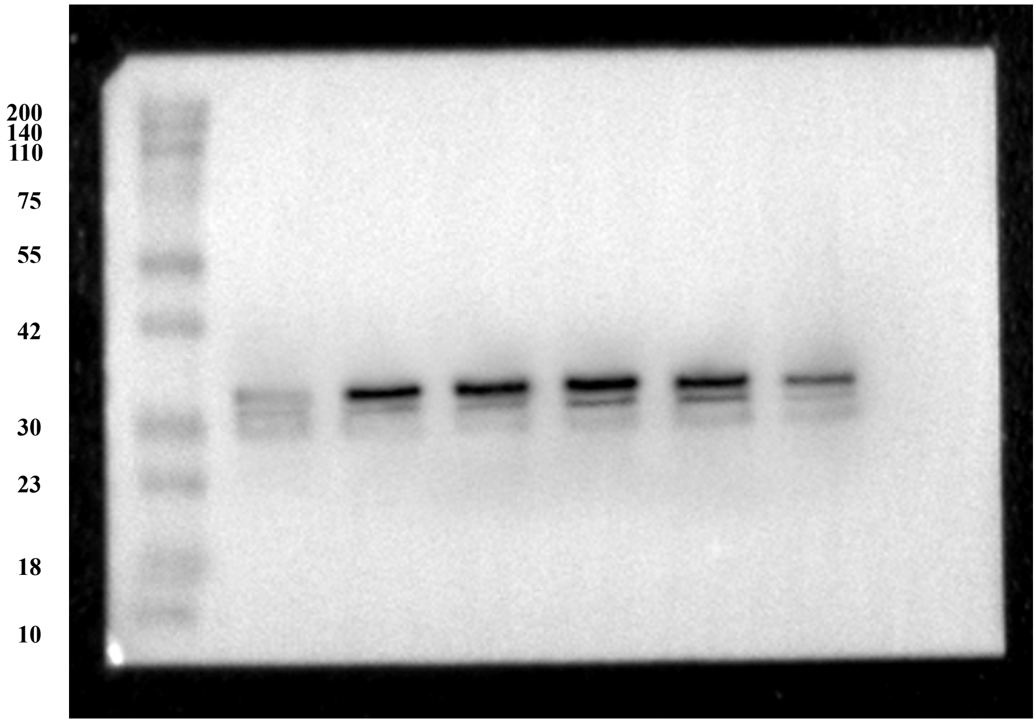

Cleaved-Caspase 3 antibody (3)

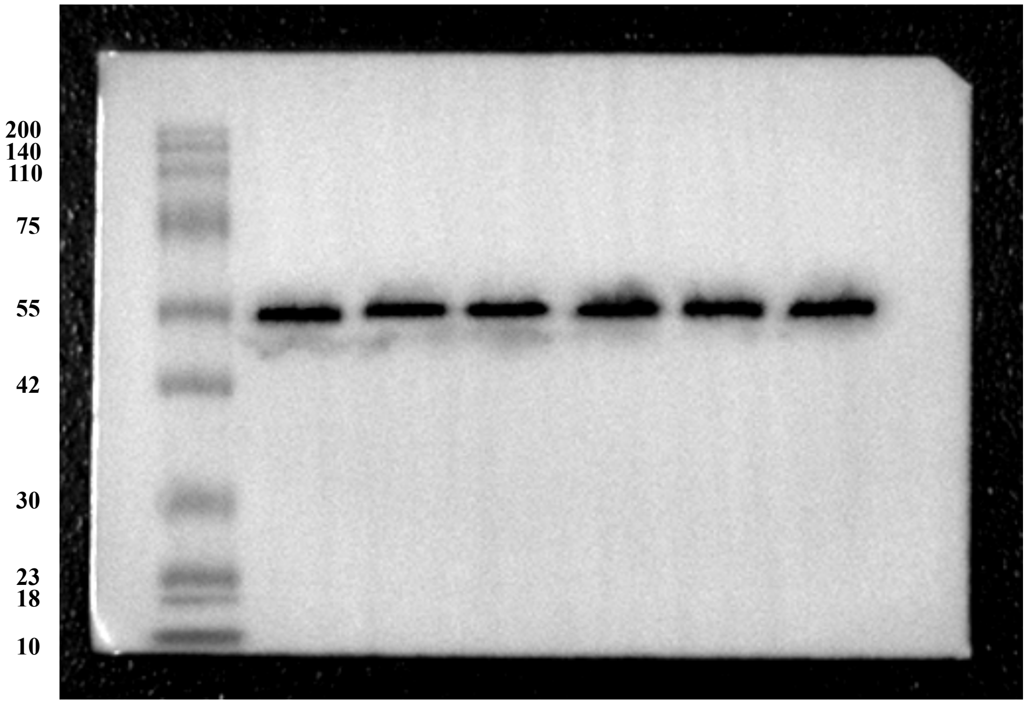

Tubulin antibody

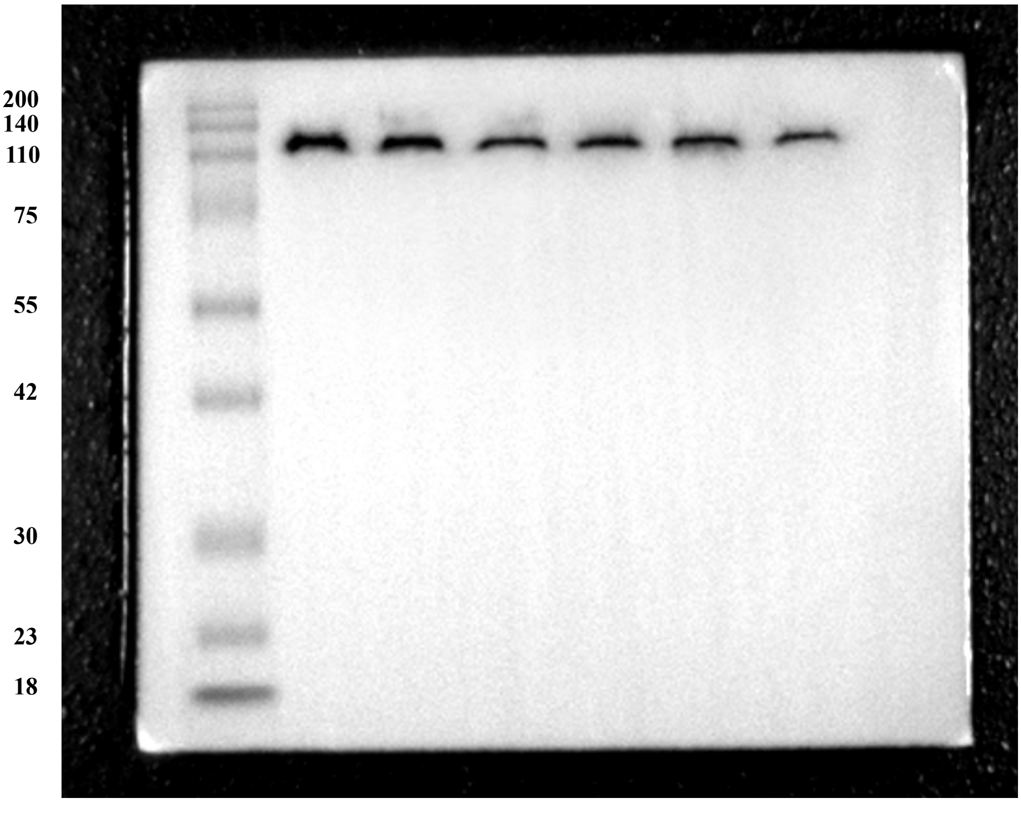

PARP1 antibody

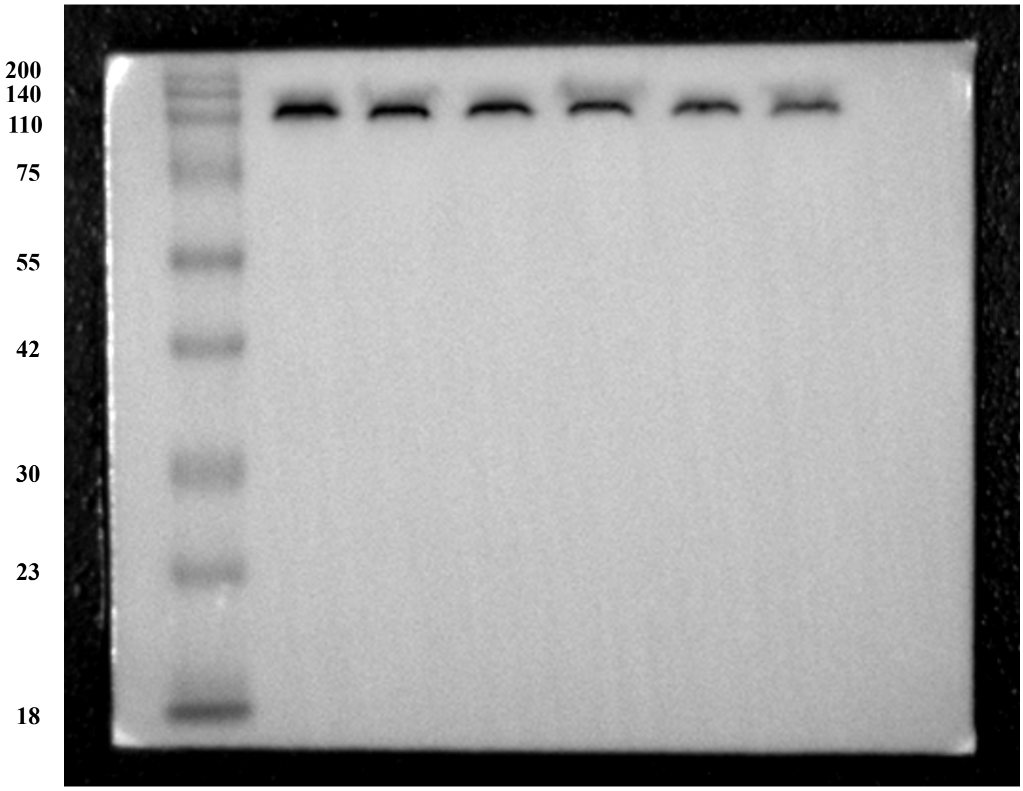

Cleaved-PARP1 antibody

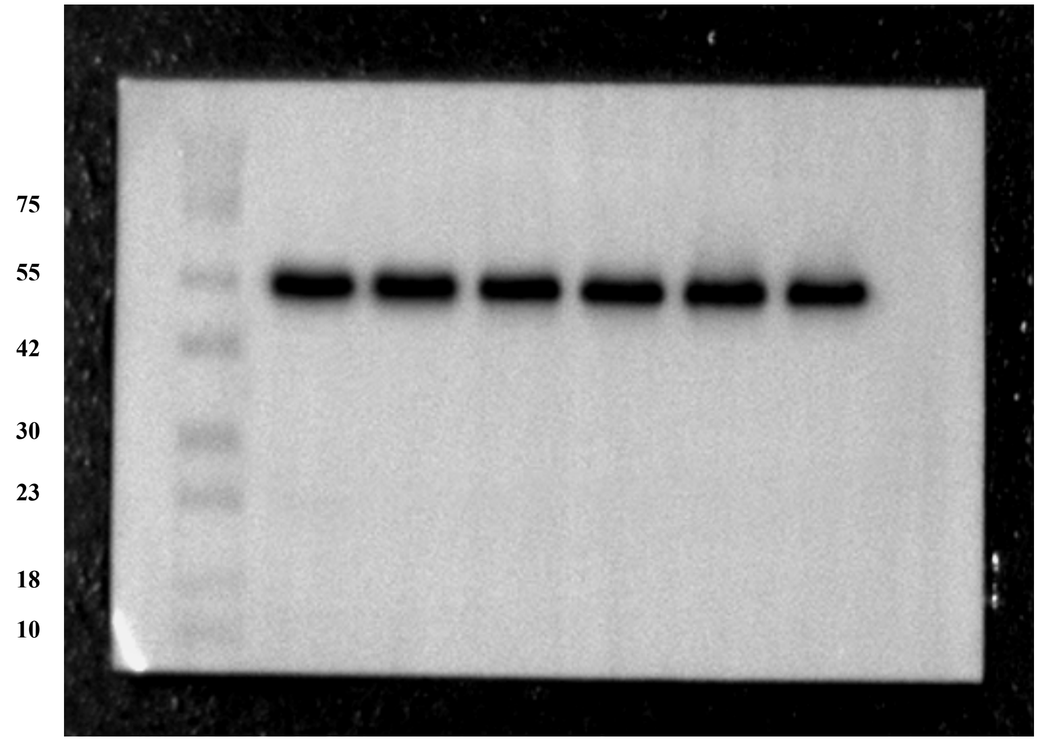

Tubulin antibody

Western Blot Fig.7B

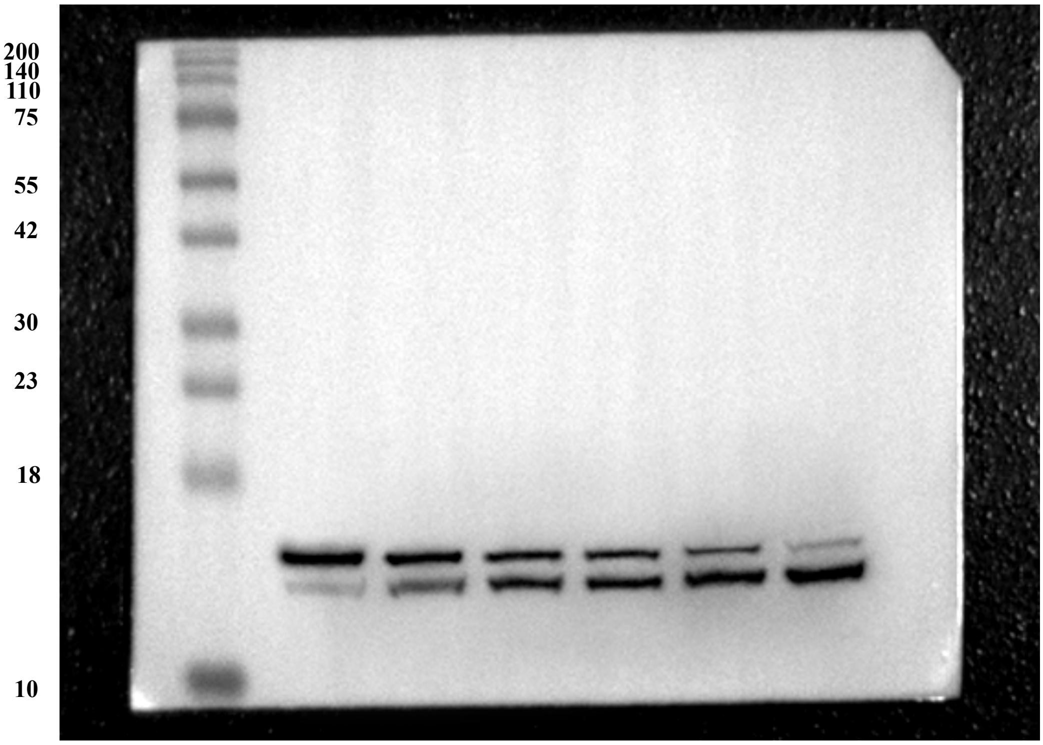

LC3 antibody

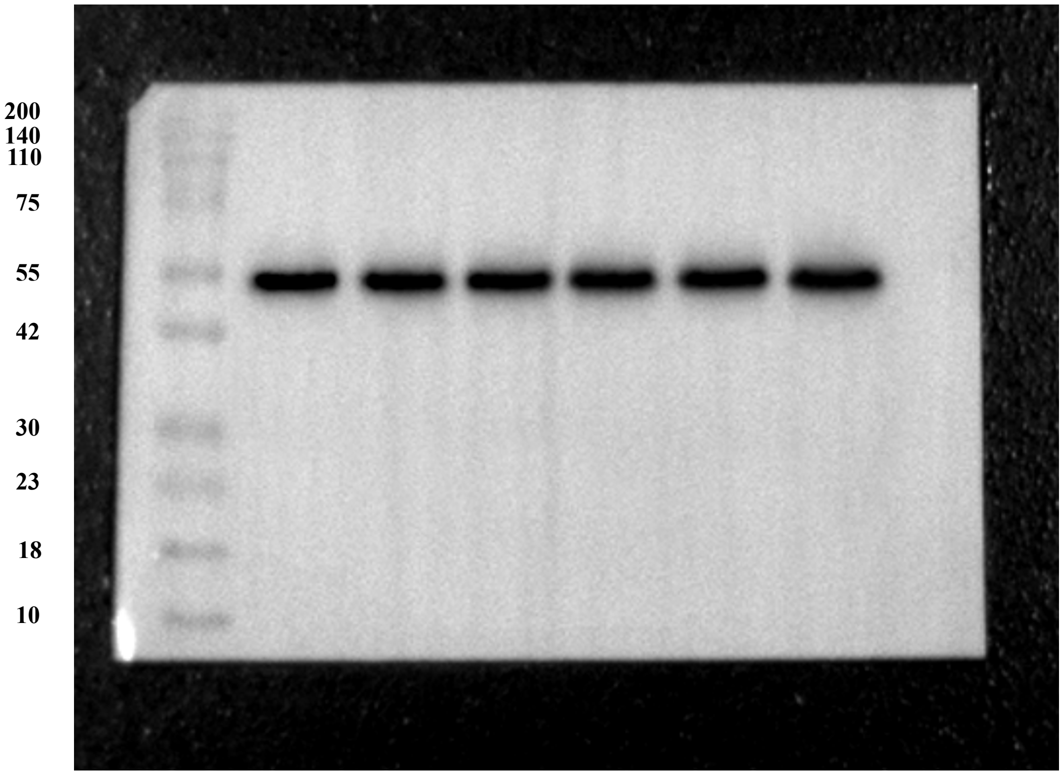

Tubulin antibody

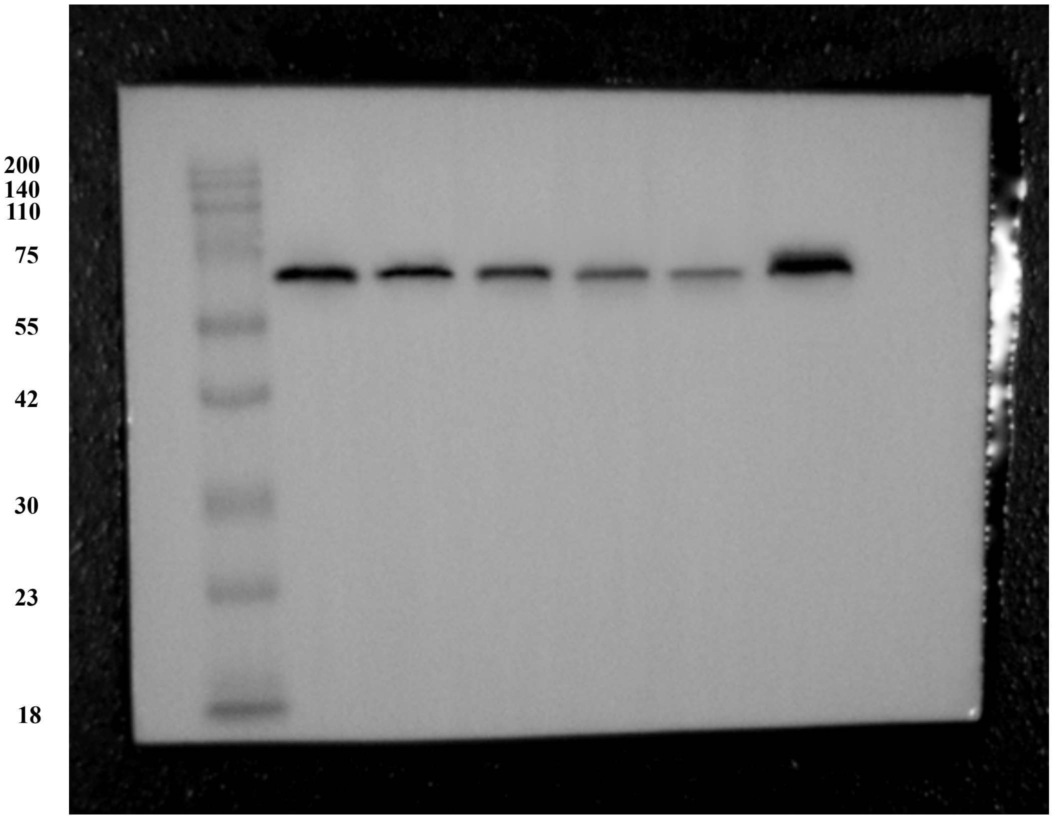

P62 antibody

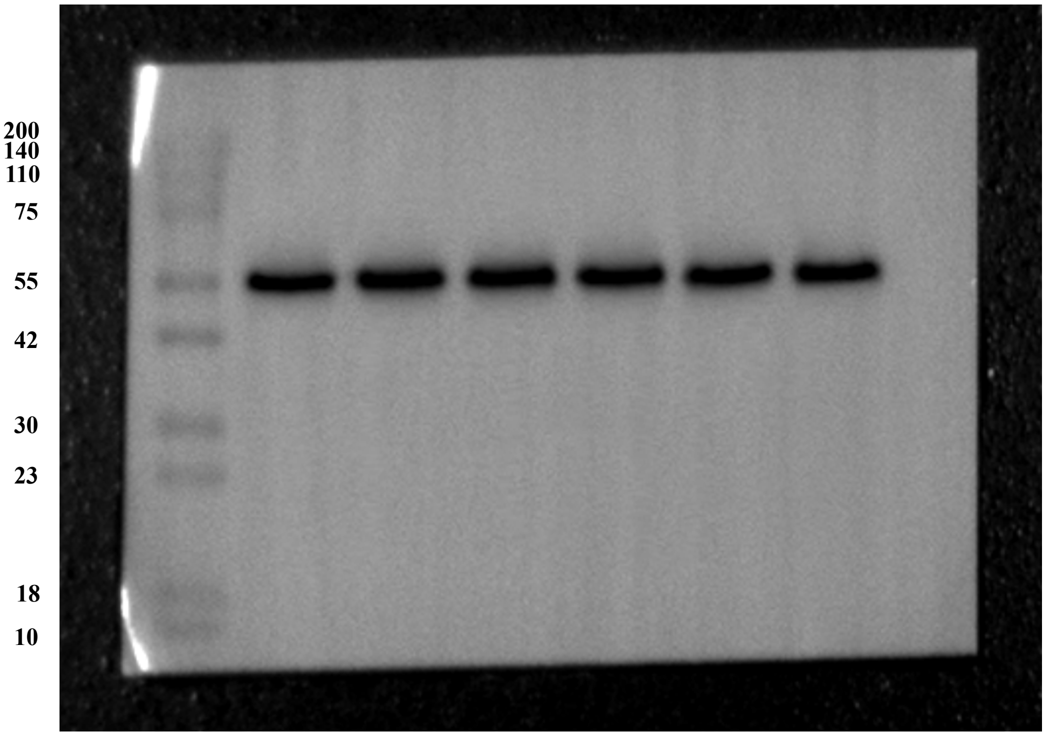

Tubulin antibody

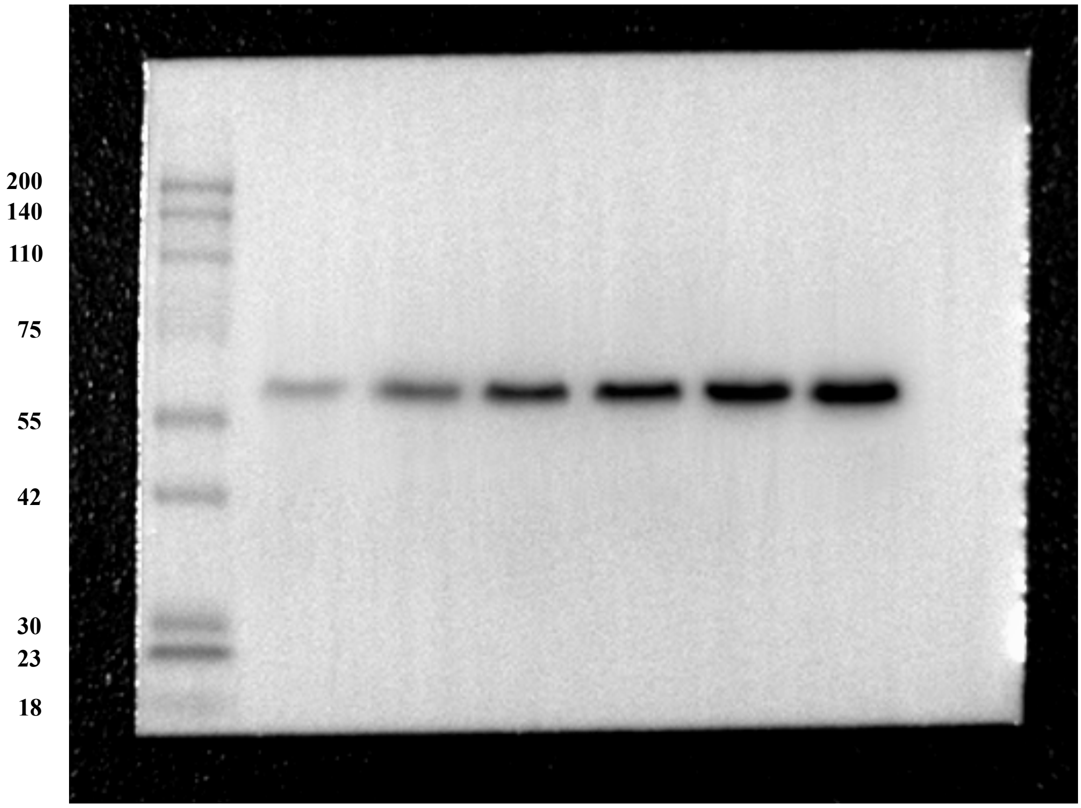

Beclin-1 antibody

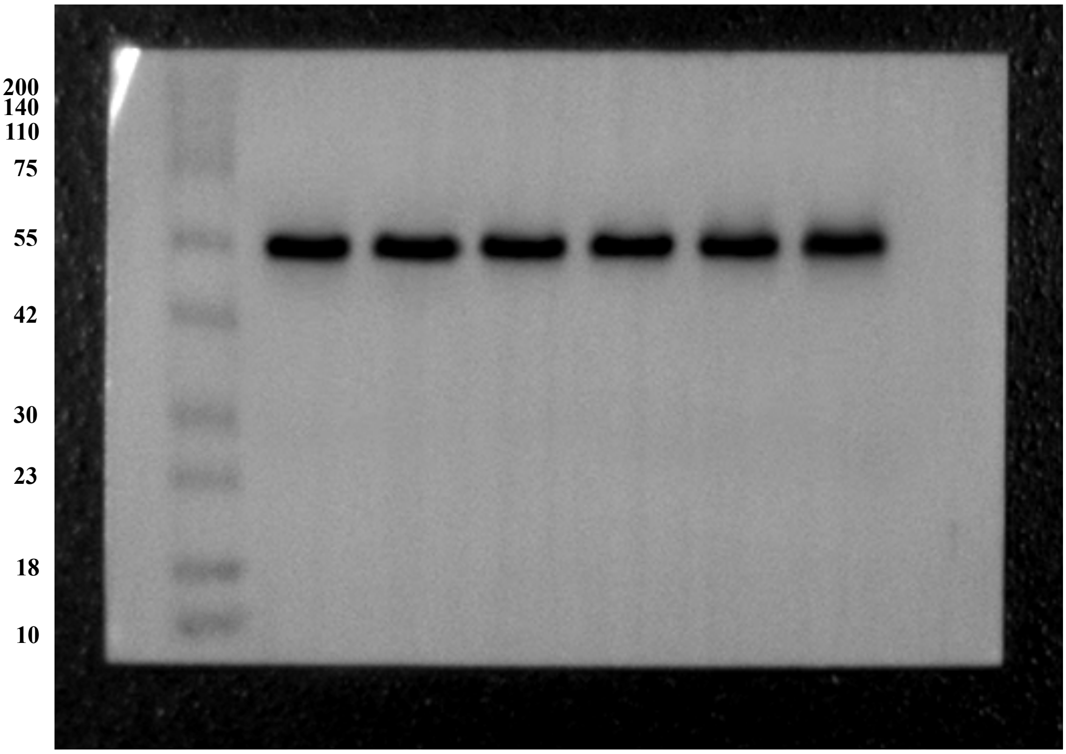

Tubulin antibody

Western Blot Fig.8A

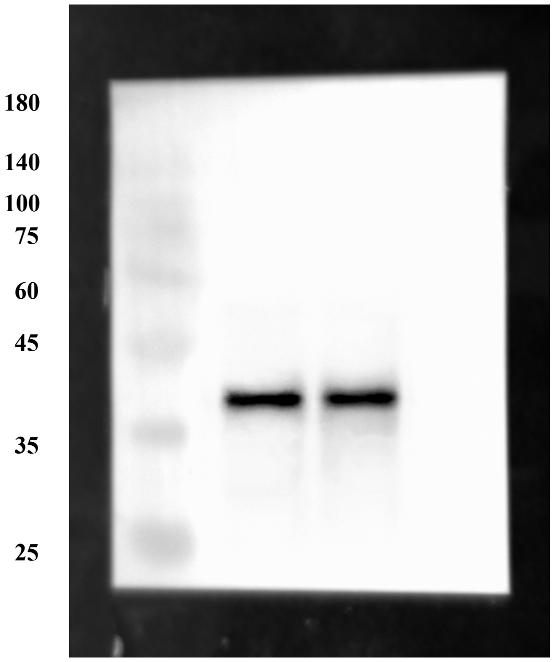

P38 antibody

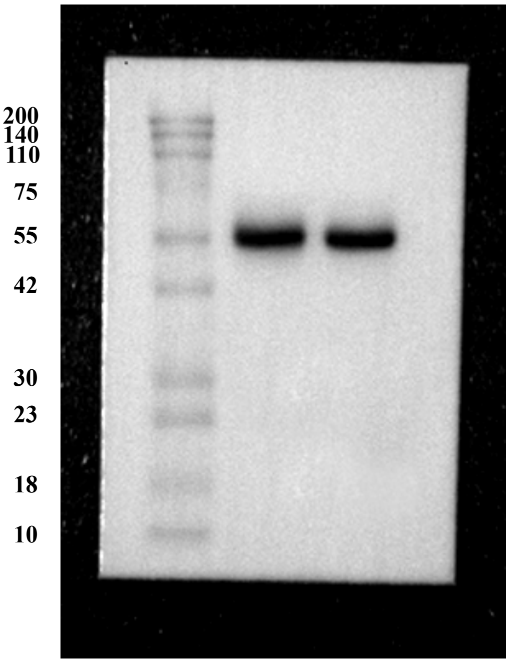

Tubulin antibody

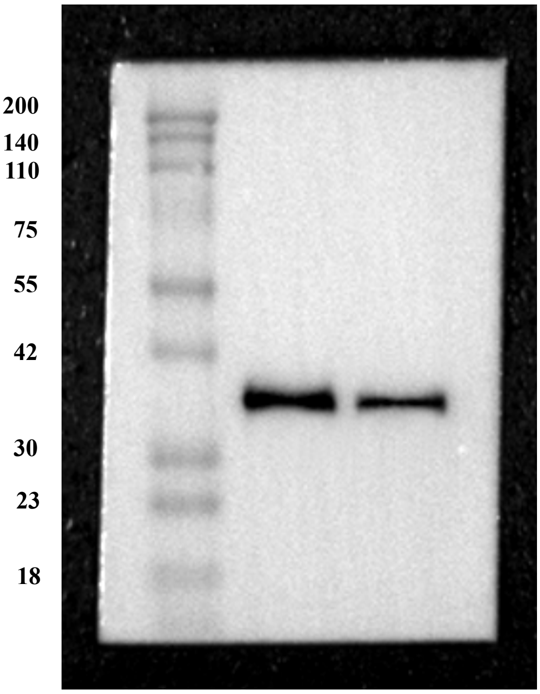

p-P38 antibody

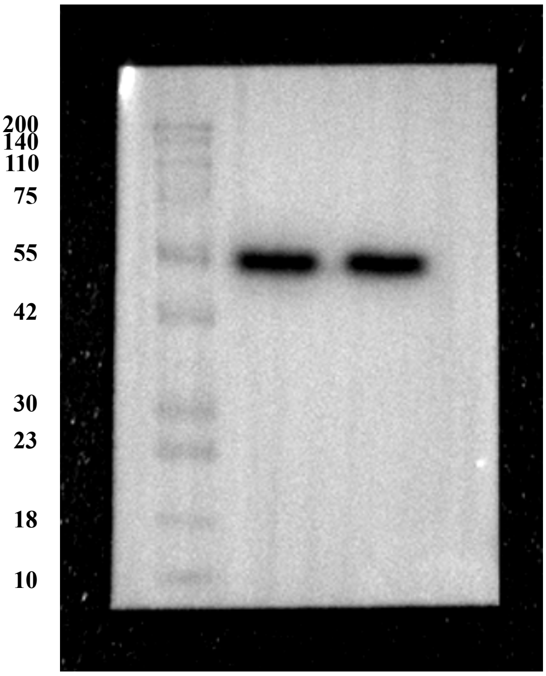

Tubulin antibody

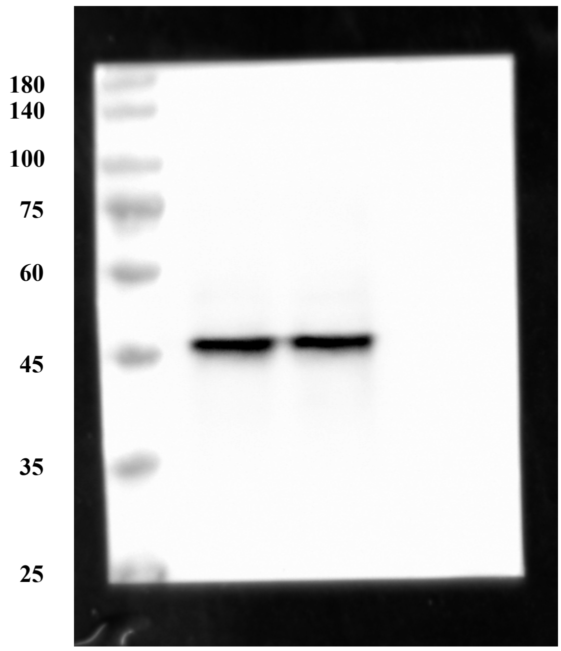

JNK antibody

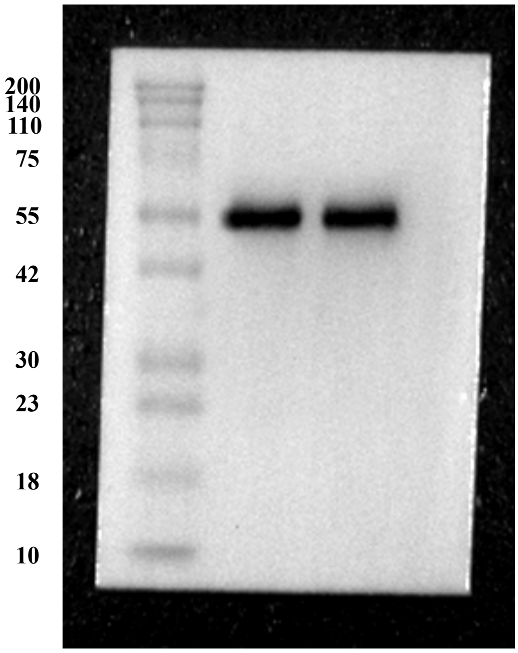

Tubulin antibody

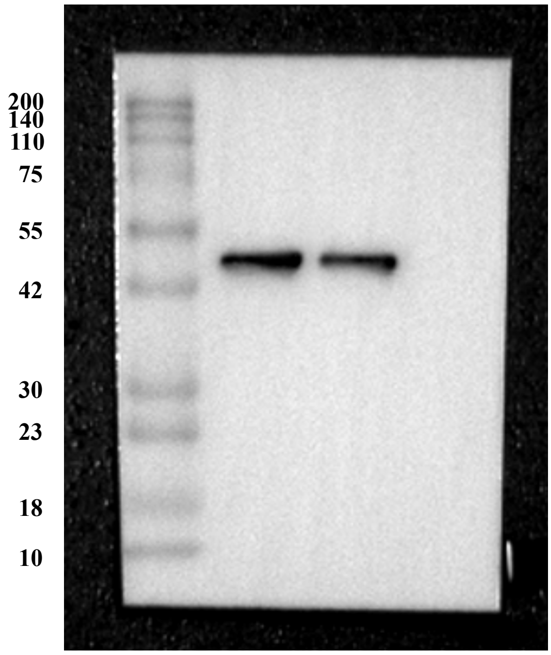

p-JNK antibody

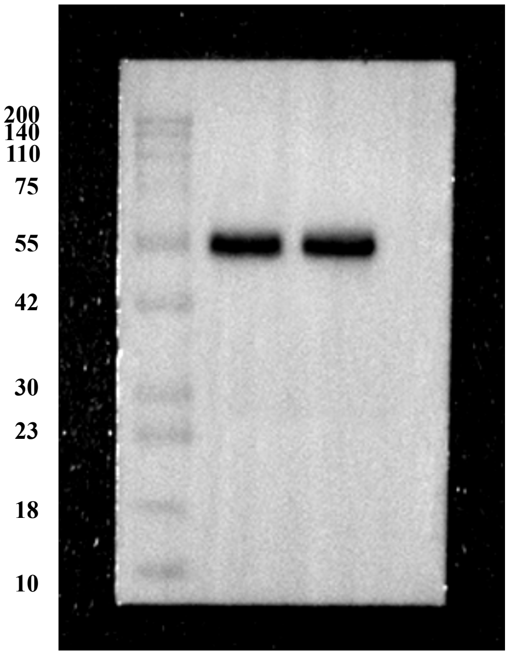

Tubulin antibody

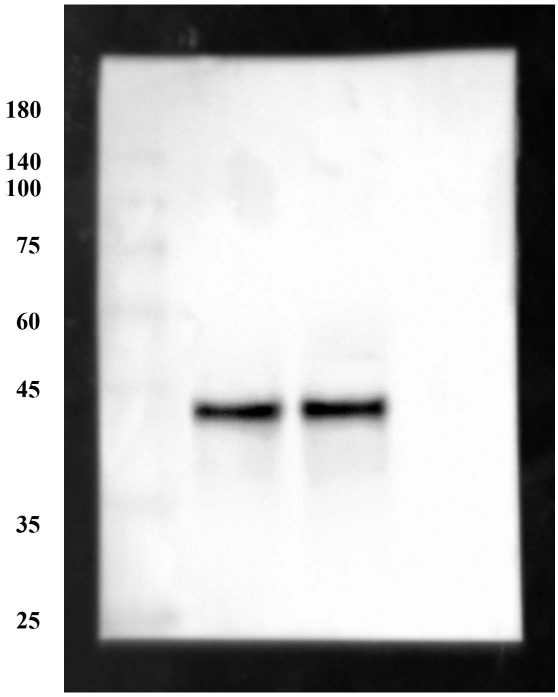

ERK antibody

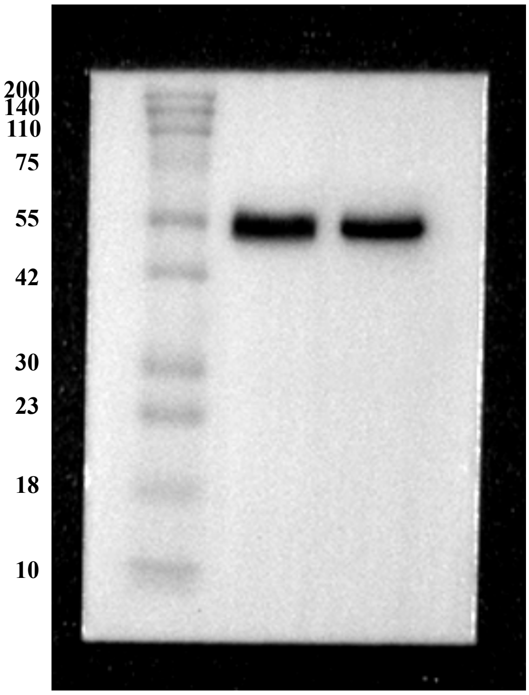

Tubulin antibody

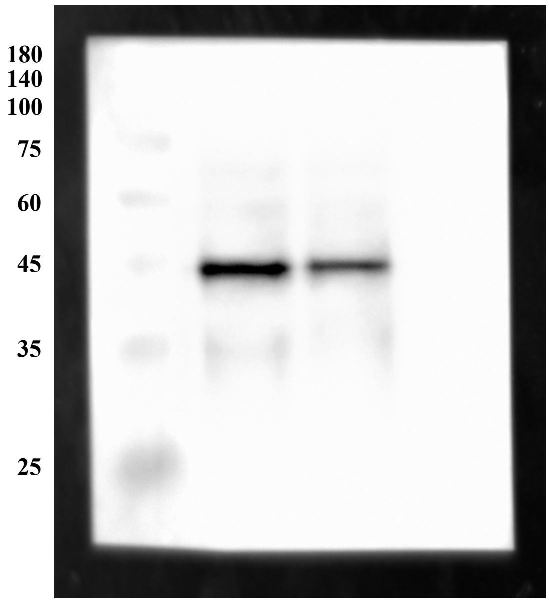

p-ERK antibody

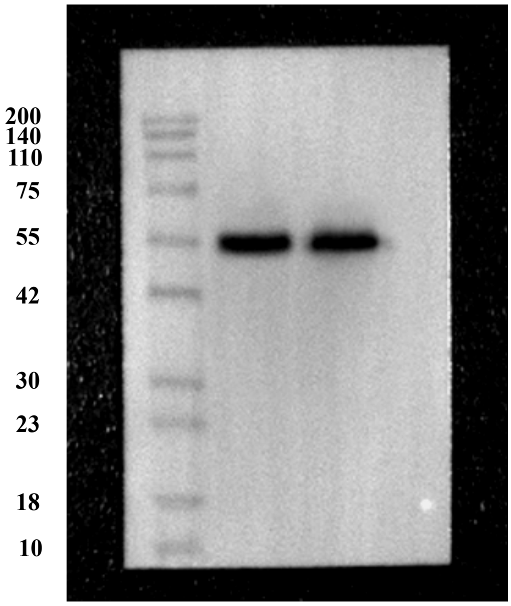

Tubulin antibody

Western Blot Fig.8B

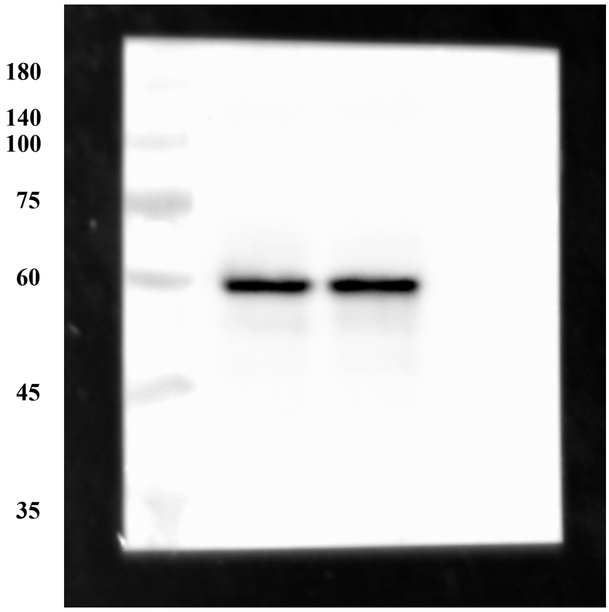

AKT antibody

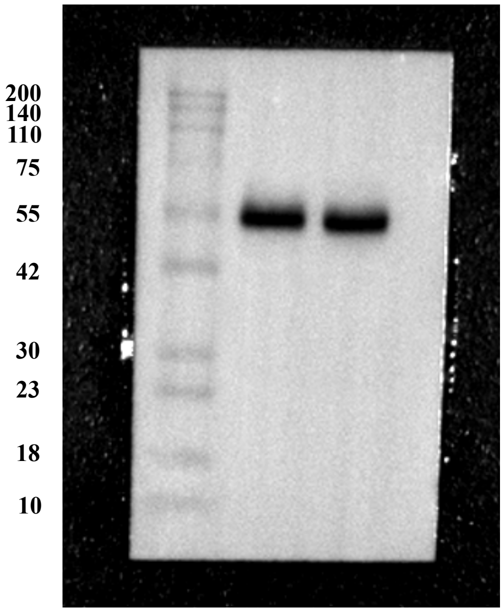

Tubulin antibody

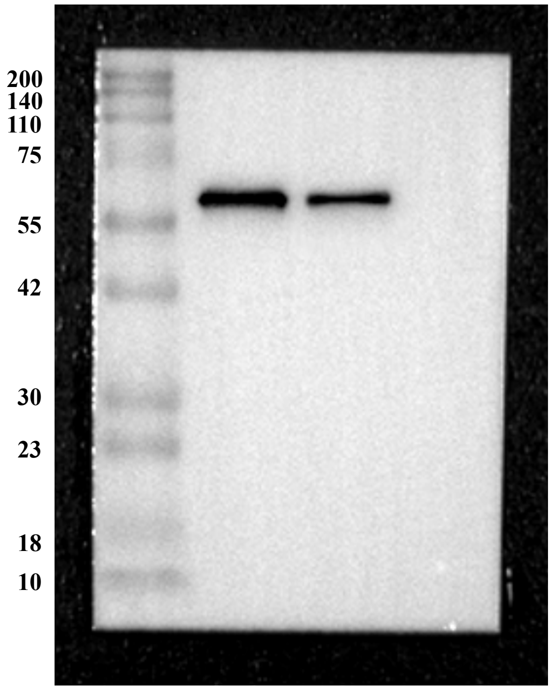

p-AKT antibody

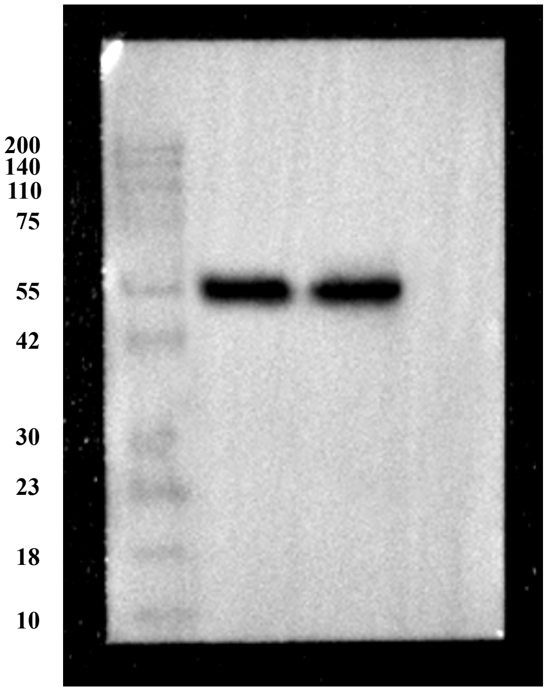

Tubulin antibody

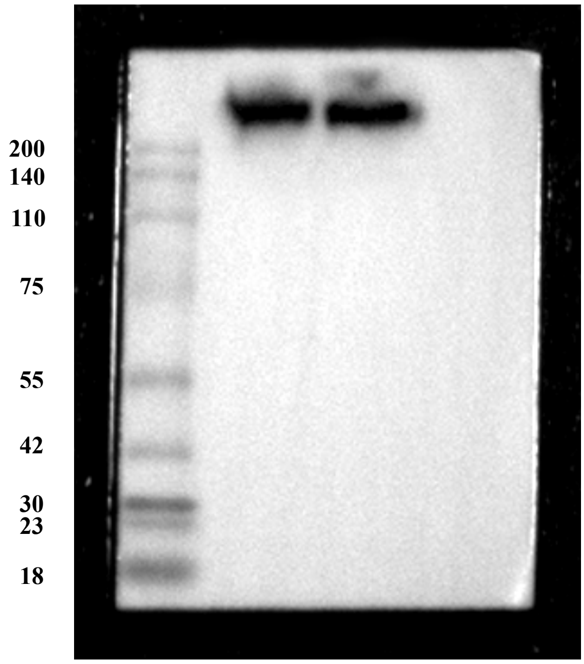

mTOR antibody

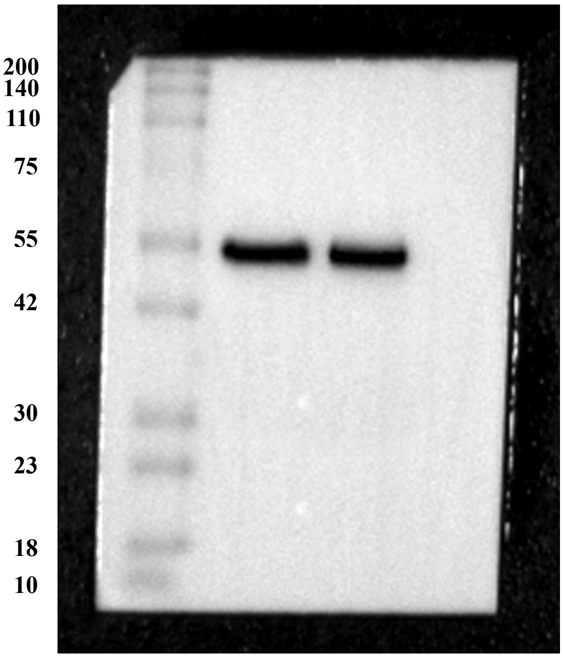

Tubulin antibody

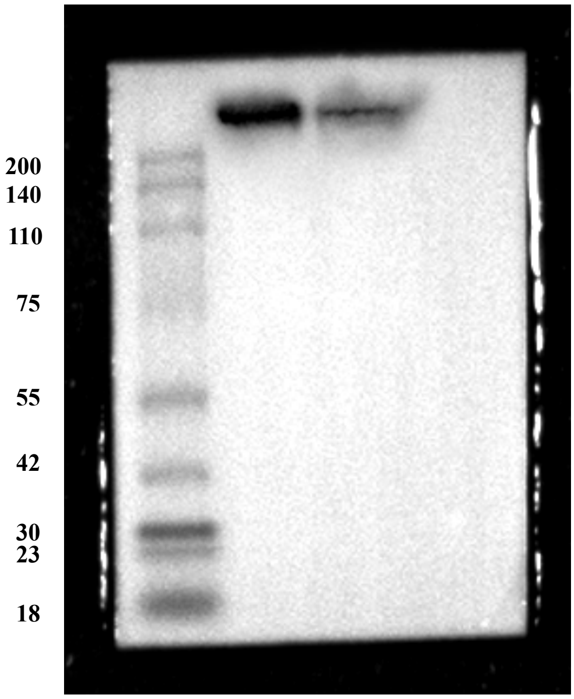

p-mTOR antibody

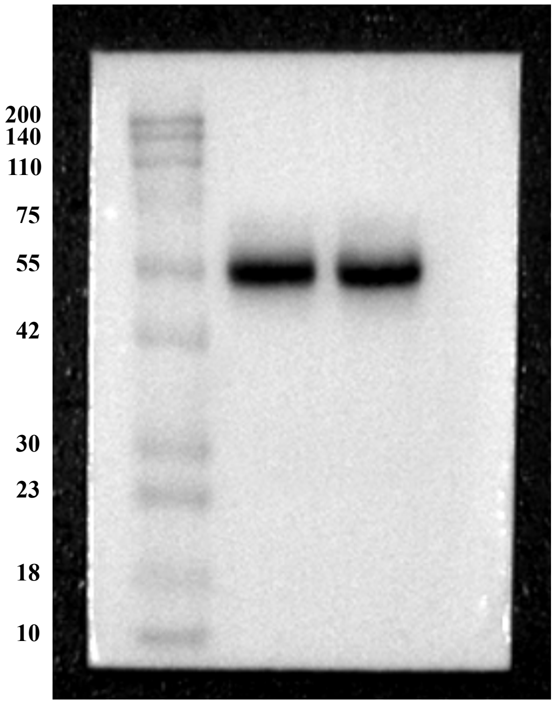

Tubulin antibody
